# DART-Eval: A Comprehensive DNA Language Model Evaluation Benchmark on Regulatory DNA

**Published:** 2025-08-04

**Authors:** Aman Patel, Arpita Singhal, Austin Wang, Anusri Pampari, Maya Kasowski, Anshul Kundaje

**Affiliations:** 1 Department of Computer Science, School of Engineering, Stanford University; 2 Department of Genetics, School of Medicine, Stanford University; 3 Department of Pathology, School of Medicine, Stanford University

## Abstract

Recent advances in self-supervised models for natural language, vision, and protein sequences have catalyzed the development of genomic DNA language models (DNALMs). These models aim to learn generalizable representations of diverse DNA elements, potentially enabling various downstream genomic prediction, interpretation and design tasks. However, existing benchmarks do not adequately assess the capabilities of DNALMs on an important class of non-coding DNA elements critical for regulating gene activity. Here, we introduce DART-Eval, a suite of representative benchmarks focused on regulatory DNA to evaluate performance of DNALMs across zero-shot, probed, and fine-tuned settings against contemporary *ab initio* models as baselines. DART-Eval addresses biologically relevant tasks including sequence motif discovery, cell-type specific regulatory activity prediction, and counterfactual prediction of regulatory genetic variants. Our systematic evaluations reveal that current annotation-agnostic DNALMs exhibit inconsistent performance and do not offer compelling gains over alternative baseline models for most tasks, despite requiring significantly more computational resources. We discuss potentially promising modeling, data curation, and evaluation strategies for the next generation of DNALMs. Our benchmark datasets and evaluation framework are available at https://github.com/kundajelab/DART-Eval

## 1 Introduction

Large Language Models (LLMs) have revolutionized natural language processing [31] by learning complex patterns, contextual relationships, and hierarchical structure within text in a self-supervised manner. In the biological domain, self-supervised language models trained on protein sequences have enabled high-performance tools for protein structure prediction and design [42, 29, 27]. Building on these advances, analogous DNA Language Models (DNALMs) have been recently developed to learn rich representations of genomic DNA sequences from one or more species, aiming to enhance DNA sequence design, functional syntax discovery, and evolutionary analyses [23].

The human genome is 3 billion base pairs of DNA and encodes two main classes of functional elements (See Appendix B for biological background). *Protein coding sequences* of approximately 20,000 genes span around 1.5% of the genome. These sequences encode a dense, information-rich syntax and serve as templates for transcription to RNA and translation to proteins. In contrast, millions of *non-coding regulatory control elements*, estimated to collectively span 5 to 20% of the genome, orchestrate complex programs of gene transcription and translation that define cell state, fate, and response [11]. The DNA sequence of transcriptional regulatory elements typically encode cell-type specific, sparse, combinatorial syntax involving short, fuzzy DNA motifs bound by regulatory DNA binding proteins (transcription factors, or TFs). The stark contrasts between coding and regulatory sequences, in their syntax, cell-type specificity, and genomic coverage, pose significant challenges for DNALMs to learn optimal representations for diverse downstream applications, necessitating a wide array of benchmarks and evaluation criteria.

Effective DNALMs must, at minimum, achieve three key objectives:

1. Learn representations that can accurately distinguish different types of functional elements.
2. Serve as foundations for training downstream supervised models using potentially sparsely labeled datasets, via probing or fine-tuning, to address salient biological questions and applications.
3. Outperform alternative *ab initio* models on biologically relevant tasks, either by themselves or as foundations for a supervised model.

The most biologically relevant applications of regulatory sequence models include interpreting and discovering functional regulatory sequences, predicting the effects of sequence perturbations including genetic variants, and designing sequences with desired properties. However, contemporary DNALMs have not been rigorously evaluated against state-of-the-art *ab initio* models for their ability to learn representations of regulatory DNA that enhance performance on these important downstream applications.

**Figure 1:**
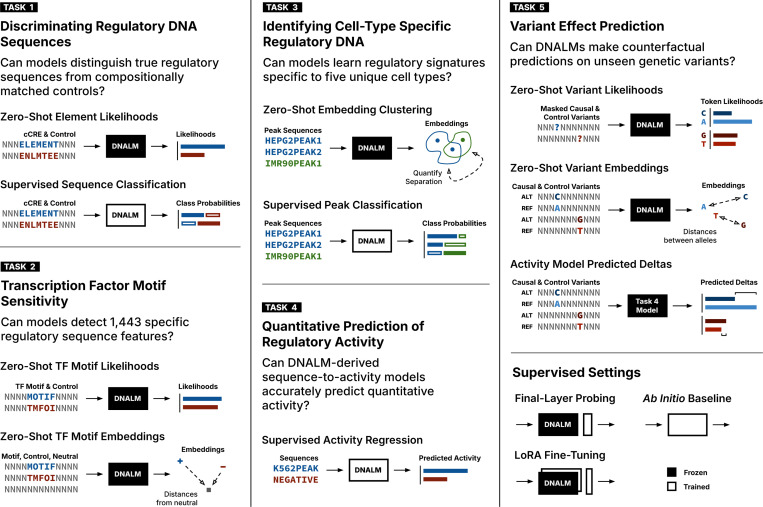
An overview of DART-Eval tasks and settings

To address this gap, we introduce DART-Eval (DNA RegulaTory Evaluations), a suite of benchmarks to assess the utility of regulatory DNA representations learned by DNALMs (Figure 1). We evaluate the performance of contemporary large DNALMs, trained genome-wide without leveraging genomic annotations, across five sets of downstream tasks of increasing difficulty, comparing their zero-shot, probed and fine-tuned performance against state-of-the-art, *ab initio* models.

DART-Eval offers:

- **Five sets of tasks with representative benchmark datasets** that enable evaluation of diverse properties and use cases of regulatory DNA representations learned by DNALMs.
- **An adaptable framework** to prepare embeddings, train probing models, and fine-tune DNALMs.

Our systematic evaluation reveals three key results:

- Embedding-free methods generally outperform embedding-based methods.
- Simpler *ab initio* supervised models match or exceed the performance of much larger, fine-tuned DNALMs.
- DNALMs perform particularly poorly on counterfactual prediction tasks and substantially underperform *ab initio* supervised models.

DART-Eval serves as a resource for developing more effective regulatory DNA models and rigorously assessing the utility of DNALMs.

## 2 Related Work

### 2.1 DNA language models of the human genome

In this work, we specifically evaluate contemporary DNALMs that are trained in an annotation-agnostic manner across the entire human genome and in some cases, additional species. We evaluate Caduceus, DNABERT-2, GENA-LM, HyenaDNA, Mistral-DNA, and Nucleotide Transformer, summarized in Table 1 [39, 47, 16, 34, 5, 13]. We select the most capable version of each model family, with resource requirements summarized in Table S5. These models employ diverse approaches. Pre-training objectives include BERT-style masking and autoregressive next-token-prediction. Tokenization strategies include single-nucleotide, non-overlapping fixed-size *k*-mers, and byte-pair encodings. Model architectures include Transformers (self-attention), Hyena (sub-quadratic-time implicit long convolutions), Mamba (selective state space), and differ in maximum context lengths.

Our current study does not include some other classes of DNALMs, such as those modeling evolutionarily distant organisms [33, 9], annotation-aware models trained on pre-specified classes of genomic regions [23], and models that incorporate additional features like multiple sequence alignments [9]. Our benchmarks can be readily adapted to these models as well, and we aim to include these in future extensions.

### 2.2 Previous DNALM benchmarks

Several DNALM benchmarks have been proposed [34, 13, 16, 47, 30, 41], including evaluations focused on regulatory DNA. However, existing evaluations face three critical limitations. First, they focus on surrogate prediction tasks that do not directly address the main downstream applications (interpretation, counterfactual predictions, design). Second, fundamental flaws in benchmark dataset design undermine evaluations. Lastly, reliance on oversimplified or flawed baseline approaches often exaggerates the relative benefits of DNALMs (summarized in Table 2).

Current benchmarks often rely on surrogate predictive tasks that do not directly address key downstream applications. For example, DNALMs are often fine-tuned to classify different classes of regulatory elements like promoters or enhancers and evaluated on the same task [13, 34, 16, 47]. However, the practical value of these models lies in their ability to reveal predictive sequence features or design synthetic sequences for each class, capabilities that most existing benchmarks fail to evaluate.

Dataset design issues, such as the lack of rigorous controls, further compromise evaluations. Regulatory DNA sequences typically have higher G/C nucleotide content than the genomic background. Without compositionally matched background sequences, classifiers may perform well without learning biologically relevant regulatory DNA sequence features [44, 26, 46, 11]. Confounders also affect counterfactual variant effect prediction tasks, where the goal is to use variant effect scores to discriminate functional genetic variants from other background variants. Many evaluations incorrectly use trait-associated variants to define functional variant sets, overlooking that most such associations are correlative rather than causal due to linkage disequilibrium (LD) [43]. Benchmarking datasets must therefore be carefully curated to control for such confounders.

The rapid progress in regulatory genomics in terms of data generation and modeling has rendered many previous cutting-edge resources obsolete. However, many benchmarks often use outdated datasets and baseline models. The latest *ab initio* supervised models are trained on quantitative, high-resolution molecular readouts of regulatory activity across multiple cell types and vastly improve upon previous generation binary classification models [11, 6, 35]. Effective benchmarks must incorporate these state-of-the-art models as baselines.

Recent benchmarks have begun addressing these limitations by incorporating high-quality regulatory profiling experiments across diverse cellular contexts [30] and experimentally validated regulatory genetic variants [41]. Our work complements these efforts with carefully curated benchmark datasets that (1) carefully control for biological confounders, (2) enable evaluation of the model’s ability to discover a comprehensive set of regulatory sequence features across diverse cell types, and (3) utilize panels of high-confidence causal variants affecting regulatory activity.

## 3 DNALM evaluation approaches and baselines

This section describes the different types of zero-shot, probed, and fine-tuned DNALM evaluation approaches as well as the baseline models.

### Zero-Shot Analyses

We used two broad strategies to evaluate pre-trained DNALMs in a zero-shot manner without task-specific tuning. First, embedding-based evaluations utilize the mean last-layer embeddings across tokens. Second, likelihood-based evaluations are derived from the cross-entropy loss as the input (interpreted as a negative log-likelihood and closely related to perplexity scores). For autoregressive models, the likelihood is computed from the overall loss of the next-token predictions. For masked models, the quasi-likelihood for a single token is defined as the likelihood of that token, given an input masked at that token. These quasi-likelihoods are then summed across tokens.

### Supervision via probing or fine-tuning

We implemented two efficient approaches for supervised tuning of the pre-trained DNALMs. First, final-layer probing involves training a CNN classifier on the final hidden layer outputs of the DNALM, with the base model frozen. Second, parameter-efficient fine-tuning uses LoRA [20] to train low-rank adapters on all linear and convolutional layers in the base model. The specific architectures used are detailed in Appendix D.2.

### *Ab Initio* Baselines

We compared the DNALMs to supervised baseline models that were trained *ab initio*. First, we used ChromBPNet as the baseline model for regression tasks (Section 4.1) involving chromatin accessibility (a measure of regulatory activity). ChromBPNet is a dilated CNN architecture with a 2 Kb local receptive field that predicts base-resolution signal profiles of chromatin accessibility from regulatory DNA sequence [35]. Second, for the other tasks (Sections 4.2 - 4.5), we used a simple CNN classifier architecturally similar to our probing CNN architecture but with one additional layer. Lastly, for the cell-type-specific sequence classification task (Section 4.3), we used a CNN model similar to ChromBPNet but trained on binary labels. Further details are provided in Appendix D.3.

## 4 Prediction tasks and evaluation results

### 4.1 Distinguishing regulatory DNA from background sequences

First, we designed a relatively easy prediction task that evaluates a model’s ability to distinguish high-confidence regulatory element sequences from compositionally matched synthetic control sequences. The positive element set was derived from 2.3 million candidate cis-regulatory elements (cCREs) curated by the ENCODE consortium based on experimental profiling of biochemical regulatory activity [12]. Negative set sequences were generated by shuffling each sequence while maintaining dinucleotide frequencies, thereby destroying syntactic information but preserving key compositional properties [36, 8].

#### Data

The dataset for this task consists of 2.3 million cCREs of length 350 bp (Appendix C.1) and an equivalent number of synthetic dinucleotide-shuffled negatives. See Appendix E.1 for preprocessing details.

#### Metrics

In the zero-shot setting, DNALM model likelihoods were obtained for each pair of cCRE and shuffled negative, with a “correct” prediction assigning a greater likelihood to the cCRE than the negative. In supervised settings, the models predict whether a given element is a cCRE or a negative. We define “absolute accuracy” as the fraction of elements and controls classified correctly, and “paired accuracy” as the fraction of pairs where the model assigns a greater probability to cCREs in the positive set than the synthetic controls in the negative set.

#### Results

All DNALMs prioritized cCREs over synthetic negative control sequences in a zero-shot setting (Table 3), suggesting that the models are likely learning at least some regulatory sequence features beyond compositional biases. Probing models demonstrated similar absolute accuracies and improved paired accuracies, compared to the zero-shot setting. Fine-tuning yielded a further improvement in prioritization, with paired accuracies approaching 1. The *ab initio* CNN baseline model, in comparison, demonstrated similar performance to the probed models.

### 4.2 Assessing sensitivity to known regulatory sequence motifs

Next, we evaluated whether the DNALMs had learned sequence features that are known to drive regulatory activity. Transcriptional regulatory elements encode one or more short sequence motifs that recruit sequence-specific regulatory DNA binding proteins called transcription factors (TFs). Here, we assess the models’ abilities to distinguish known TF binding motifs from matched shuffled negative control motifs.

#### Data

We individually tested known consensus binding motifs of 1443 TFs from the HOCOMOCO v12 database [45], further described in Appendix C.2. We derived 100 neutral backgrounds from dinucleotide-shuffled ENCODE cCRE sequences. Positive sequences were constructed by inserting TF motifs at the center of each background sequence. Negative sequences were similarly constructed by inserting shuffled motifs. Both forward and reverse complements of each positive and negative sequence were scored through the models and used for evaluation.

#### Metrics

We evaluated the DNALMs in a zero-shot setting by calculating likelihoods and embeddings. For the likelihood-based approach, we considered a prediction to be “correct” if the model assigned a higher likelihood to a positive sequence than its corresponding negative sequence. For the embedding-based approach, we defined a correct prediction as one where the cosine embedding distance from the neutral background sequence to its corresponding positive sequence was greater than the distance from the neutral sequence to a corresponding negative sequence. Accuracies were computed individually for each TF motif (Appendix E.2).

#### Results

**Figure 3:**
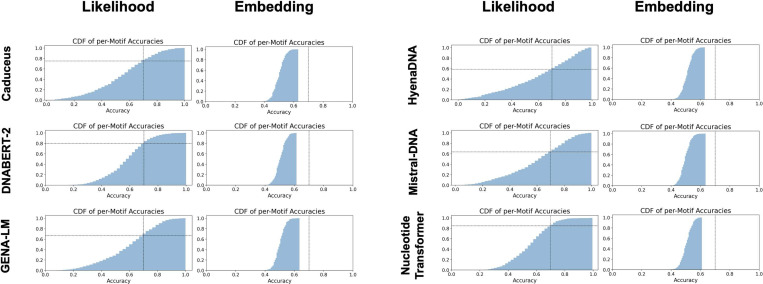
Distributions of zero-shot accuracies across 1443 transcription factor motifs, testing the ability to distinguish motif instances from background sequences. Vertical and horizontal lines represent 70% accuracy thresholds. In the likelihood setting, models identify most but not all motifs. In the embedding settings, models fail to distinguish motifs from background sequences.

In the likelihood setting, all DNALMs were able to prioritize positive sequences containing TF motifs over negative controls (Figure 3). However, accuracies varied substantially across motifs, suggesting that some motifs are likely not encoded in the internal representations learned by the DNALMs (Table S7). Notably, motif likelihood scores were highly correlated between models, suggesting a strong influence of the underlying pre-training data distribution on performance (Figures S2, S3). These trends were also preserved when motifs were aggregated over TFs belonging to the same families based on the similarity of their DNA binding domains and motifs (Figures S4, S5). In contrast, no model reliably prioritized motifs in the embedding setting, with the vast majority of accuracies falling between 40% and 60% (Figure 3). These results indicate that final-layer averaged embeddings and cosine distance measurements do not fully capture the models’ expressivity. Lastly, motif discovery performance (this task) was nearly always lower than performance for discriminating regulatory elements (Section 4.1). We hypothesize that this discrepancy stems from two key factors. First, DNALMs learn only a subset of TF motifs, primarily capturing those that appear frequently across the genome. Second, regulatory elements contain multiple TF binding sites, allowing successful discrimination even with an incomplete repertoire of learned motifs.

### 4.3 Learning cell-type-specific regulatory sequence features

Cell-type identity emerges from distinct patterns of regulatory element activity across a shared genome. Hence, we evaluated whether representations learned by DNALMs encode cell-type specific regulatory sequence features.

#### Data

We curated cell-type specific regulatory elements across five diverse cell lines based on ATAC-seq chromatin accessibility experiments that highlight regulatory regions bound by TFs ([10]). Specifically, we used regions with strong ATAC-seq accessibility signal (peaks) as candidate regulatory elements in each cell line C.3. We then used DESeq2 for differential analysis to identify cell-type specific elements that showed significantly higher chromatin accessibility in exactly one cell type relative to the others [28] (Appendix E.3).

#### Metrics

We evaluated models in zero-shot and supervised settings. For zero-shot evaluation, we clustered model embeddings of the cell-type specific regulatory sequences using *k*-means and quantified label separation using the adjusted Mutual Information Score across labels [1]. In the supervised setting, we evaluate the performance of classification of the cell-type specific regulatory sequences using overall accuracy and binary classification metrics (accuracy, AUROC, AUPRC) for each cell type versus the others (Appendix E.3).

#### Results

**Figure 4:**
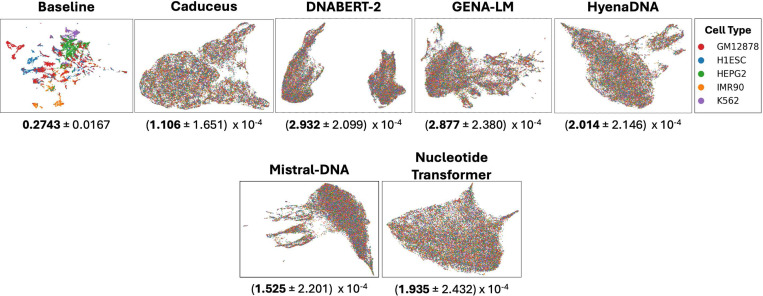
UMAP of model embeddings for sequences with experimentally identified cell-type-specific activity, colored by the true labels. The baseline embedding is a vector of canonical motif instances identified by FIMO. Numerical values are adjusted mutual information scores between true labels and a *k*-means clustering with *k* = 50, along with a 95% confidence interval across clustering seeds, measuring the clustering quality w.r.t. the true labels. Only the baseline model yielded a useful embedding space for distinguishing sequence features for cell-type-specific activity.

In the zero-shot setting, all DNALMs showed poor cell-type separation in their embeddings when compared to a simple baseline approach that used *k*-means on the sequences represented using motif counts of known motifs (Figure 4) (Appendix E.3). In the supervised setting, the *ab initio* CNN baseline generally matched or outperformed all other models. Fine-tuned models outperform probed models, which performed comparably to a smaller *ab initio* CNN baseline (Tables 4, S8 - S13).

### 4.4 Predicting quantitative measures of regulatory activity from sequence

ATAC-seq and DNase-seq experiments performed in a cell type of interest provide genome-wide quantitative measurements of chromatin accessibility [10, 40]. The quantitative chromatin accessibility signal of a regulatory element is dictated by the repertoire of TFs that bind specific syntax of sequence motifs encoded in its sequence. We therefore evaluated whether representations learned by DNALMs enable accurate prediction of quantitative chromatin accessibility.

#### Data

We trained sequence-to-activity (S2A) regression models to predict quantitative DNase-seq signal (measured as log(counts of sequencing reads)) over 2 Kb genomic sequences. The input sequences included DNase-seq peaks (regions with a strong, statistically significant signal) and other G/C-content-matched background genomic regions. Models were trained and evaluated separately on DNase-seq data from five cell types as in Section 4.3 (Appendix C.3).

#### Metrics

We assessed regression performance using Pearson and Spearman correlation between predicted and observed signal, evaluated across peak regions only and across a union of peak and background regions. Additionally, we computed binary classification metrics (AUROC and AUPRC), using a positive set of high-confidence, reproducible peaks and a negative set of background regions (Appendix E.4).

#### Results

Fine-tuned DNALM S2A models showed strong performance, with the strongest models matching or moderately outperforming the *ab-initio* baseline CNN (ChromBPNet) model on regression and classification metrics (Tables 5, S14 - S18). In contrast, probed models substantially underperformed ChromBPNet, indicating that frozen last-layer embeddings lack sufficient expressivity for accurate prediction of quantitative accessibility.

### 4.5 Predicting counterfactual effects of regulatory genetic variants

A critical challenge in human genetics is predicting how genetic variants affect gene regulation through changes in chromatin accessibility. Models trained to predict regulatory activity from sequence (S2A models) (such as those in Section4.4) are typically used in a counterfactual setting to predict the effects of genetic variants on regulatory activity. This is a particularly challenging task since the S2A models are never directly trained on genetic variation data. We evaluated the ability of DNALMs to prioritize and predict the quantitative effects of regulatory genetic variants that impact chromatin accessibility.

#### Data

We utilized data from two quantitative trait locus (QTL) mapping studies that associate genetic variation with variation of chromatin accessibility from ATAC-seq or DNase-seq experiments across a large cohort of lymphoblastoid cell lines (LCLs) from individuals of African ancestry [15, 14] (Appendix C.4). These datasets identify genetic variants with statistically significant effects on chromatin accessibility as caQTLs (for ATAC-seq) and dsQTLs (for DNase-seq). We enrich for likely causal caQTLs and dsQTLs by restricting to those that fall within accessible regulatory elements (peak regions). These variants form the positive set. The negative set consists of other background genetic variants in peak regions that do not exhibit statistically significant associations with chromatin accessibility. Each genetic variant consists of a pair of alleles (two different nucleotides) ∈ {A, C, G, T} and a label *y* ∈ {0,1} with 0 = background and 1 = significant.

#### Metrics

We extracted the 2 Kb genomic sequence context of each variant and scored two versions of the sequence that contain each of the alleles of the variant with different models. We scored variants in both zero-shot and supervised settings. For embedding-based zero-shot scoring, we computed the cosine distance between the two variant allele sequences. For likelihood-based zero-shot scoring, we computed the log-likelihood difference using an input sequence with the variant-containing token masked and taking the loss with respect to the two alleles. The likelihood-based evaluations were only conducted for models with fixed encodings (Nucleotide Transformer and HyenaDNA) since changing a single base can affect the entirety of a byte-pair encoding. For supervised scoring, we used the chromatin accessibility S2A models from Section 4.4 trained on a single LCL sample (GM12878). We computed the absolute difference in predicted accessibility between the two alleles. For each dataset and variant effect score, we computed the AUROC and AUPRC with respect to the positive and negative variant sets. In addition, we computed the Pearson correlation between the reported effect size of association from the QTL study and the predicted activity difference for the supervised models (Appendix E.5).

#### Results

In the zero-shot setting, Nucleotide Transformer achieved the best performance for both embedding and likelihood-based approaches (Table 6). In supervised evaluation, fine-tuned sequence-to-activity models substantially outperformed their probed counterparts. However, despite matching ChromBPNet’s performance in chromatin accessibility prediction (Section 4.4), fine-tuned models underperformed the *ab-initio* baseline ChromBPNet in variant effect prediction. This discrepancy highlights the critical importance of including counterfactual tasks in evaluations alongside observational assessments (Tables 6, S19, and S20).

## 5 Discussion

In this study, we present DART-Eval, a suite of representative benchmark datasets for evaluating regulatory DNA representations learned by DNALMs. Our evaluations spans five tasks, comparing state-of-the-art DNALMs in zero-shot, probed, and fine-tuned settings against strong *ab initio* baseline models. The tasks increase in difficulty from detecting regulatory sequences, to regulatory motif discovery, quantitative prediction of regulatory activity, and finally counterfactual prediction of regulatory genetic variants. While DNALMs excel at simpler tasks, their performance deteriorates with increasing task complexity, highlighting the need for rigorous evaluations to accurately assess the capabilities of these models.

Although DNALMs successfully discriminate regulatory DNA from background sequences, they appear to learn incomplete repertoires of regulatory sequence features. This limitation likely stems from the sparsity and the uneven distribution of regulatory features; regulatory elements constitute only 10–20% of the human genome, and certain classes of regulatory features occur at substantially different frequencies. Potential strategies to address this challenge include balanced sampling of training examples across different classes of functional elements, incorporating regulatory annotations as tokens, or training on subsets of the genome that are functionally related (e.g., sets of candidate regulatory elements) rather than across the entire genome.

Our analysis reveals several critical insights about DNALM architecture and modeling choices. Consistent with previous studies, we observe that embedding-derived approaches (e.g. embedding distance, final-layer probing) generally underperform methods that leverage models’ full expressivity (e.g. likelihoods, fine-tuning) [32, 13]. For likelihood-based methods, masked objectives appear to be less efficient than autoregressive objectives due to required iterative token masking. Byte-pair encodings pose a particular challenge for variant effect prediction, as single-base changes can alter multiple tokens in unpredictable ways, making it difficult to compare sequence likelihoods. While fine-tuning generally achieves superior performance relative to probing, it demands significantly more computational resources, requiring gradient backpropagation through the entire model.

While DART-Eval offers a rigorous framework for the evaluation of regulatory DNA representations learned by DNALMs, future extensions could enhance its scope. Our current evaluations are limited to tasks involving short, local sequence contexts, and do not encompass tasks that require long-range context, such as the prediction of distal regulatory interactions, gene expression, or 3D genome architecture. With continued improvement in functional annotation of genomes of other model organisms, benchmarking DNALMs on diverse species will become increasingly relevant for assessing the generalizability of learned representations. Expanding task diversity to cover a more comprehensive range of regulatory elements (e.g. 5’-UTRs, 3’-UTRs, and splice sites), and incorporating evaluations related to transcriptional and post-transcriptional regulatory mechanisms would enable a more complete assessment of regulatory function coverage.

Overall, DART-Eval establishes a foundational benchmark for assessing DNALMs, with the potential to drive future advances in the development of DNALMs and their applications for regulatory genomics.

## Supplementary Material

Supplement 1

## Figures and Tables

**Figure 2:**
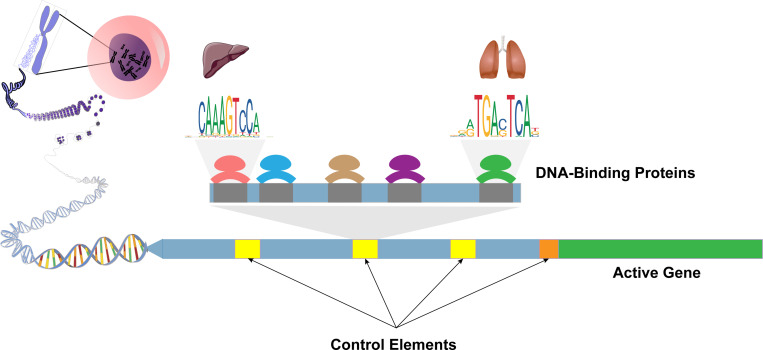
Regulatory DNA syntax is sparse, combinatorial, and cell-type-dependent [4, 3, 2, 38]

**Table 1:** An overview of evaluated DNALMs and *ab initio* baselines.

Model	Variant	Objective	Tokenization	Parameters	Species	Max Context
Caduceus	ps_131k_d-256_n-16	Masked	Single nucleotide	7.7M	Human	131 kbp
DNABERT-2	117M	Masked	Byte-pair	117M	Multi-Species	10 kbp^*^
GENA-LM	bert-large-t2t	Masked	Byte-pair	336M	Multi-Species	4.5 kbp
HyenaDNA	large-1m	Autoregressive	Single nucleotide	6.6M	Human	1 mbp
Mistral-DNA	v1–1.6B-hg38	Autoregressive	Byte-pair	1.6B	Human	10 kbp
Nucleotide Transformer^†^	v2–500m-multi-species	Masked	*k*-mer	500M	Multi-species	12 kbp

*Ab initio* Probing-head-like	-	Supervised	Single nucleotide	68K	Human	500 bp^*^
*Ab initio* ChromBPNet-like	-	Supervised	Single nucleotide	5.6M	Human	500 bp^*^
*Ab initio* ChromBPNet	-	Supervised	Single nucleotide	6.6M	Human	2114 bp^‡^

* Maximum evaluated context length. Infinite in theory.

† Abbreviated as “NT” for conciseness when necessary.

‡ Constrained by base-pair-resolution prediction head.

**Table 2:** Limitations of existing non-coding regulatory DNA evaluations for DNALMs

	GENA-LM	DNABERT-2 GUE	Nucleotide Transformer	HyenaDNA	BEND	Tang *et al.*	DART-Eval
Includes distal regulatory elements	✘	✔	✔	✔	✔	✔	✔
Number of TFs individually analyzed	3^†^	10	-	-^†^	-	10	1443
Regression tasks for quantitative assays	✘	✘	✘	✘	✘	*	✔
Compositionally-controlled negatives	✔	*	*	*	✘	✘	✔
Accounts for LD among variants	-	-	✘	-	✘	✔	✔

For each evaluation, we consider only tasks for non-coding regulation in mammalian species. We find that current evaluations focus on surrogate tasks, often draw biological conclusions using incorrect approaches, and compare with flawed baselines.

* (✘), (✔), and () indicate that a criterion is met, not met, and partially met (i.e. for only certain tasks) respectively.

(−) indicates that an evaluation has no relevant tasks for the given criterion.

† Models were fine-tuned on ChIP-Seq data from 160 TFs. However, reported statistics on individual TFs were more limited or absent.

**Table 3:** Regulatory element identification

	Zero-Shot	Probed		Fine-Tuned		Trained *ab initio*	
Model	Accuracy	Absolute Acc.	Paired Acc.	Absolute Acc.	Paired Acc.	Absolute Acc.	Paired Acc.
Caduceus	**0.971**	0.726	0.896	0.903	0.971	-	-
DNABERT-2	0.876	0.847	0.943	0.913	0.973	-	-
GENA-LM	0.947	**0.887**	**0.959**	0.909	0.972	-	-
HyenaDNA	0.891	0.847	0.935	0.877	0.952	-	-
Mistral-DNA	0.863	0.759	0.859	0.817	0.905	-	-
Nucleotide Transformer	0.745	0.819	0.917	**0.920**	**0.976**	-	-

*Ab initio* Probing-head-like	-	-	-	-	-	0.846	0.932

The best-performing model in each setting is bolded. Here, we evaluate the models’ abilities to prioritize curated regulatory elements against matched control sequences. Zero-shot accuracy and supervised paired accuracy quantify the fraction of positives prioritized over their corresponding negatives. Supervised absolute accuracy quantifies the fraction of labels assigned correctly. For this task, (1) DNALMs were effective in a zero-shot setting, (2) final-layer DNALM embeddings offered little additional signal over the raw sequence, and (3) fine-tuned DNALMs offered a moderate improvement over *ab initio* models.

**Table 4:** Cell-type-specific element classification.

		Overall	GM12878	H1ESC	HEPG2	IMR90	K562
Setting	Model	Accuracy	AUROC	AUROC	AUROC	AUROC	AUROC
Probed	Caduceus	0.281	0.535	0.622	0.680	0.576	0.587
	DNABERT-2	0.371	0.652	0.757	0.762	0.691	0.691
	GENA-LM	0.383	0.627	0.787	0.773	0.714	0.693
	HyenaDNA	0.587	0.849	0.889	0.862	0.882	0.799
	Mistral-DNA	0.329	0.582	0.678	0.723	0.643	0.646
	Nucleotide Transformer	0.420	0.744	0.795	0.783	0.779	0.711

Fine-Tuned	Caduceus	**0.671**	0.900	**0.937**	**0.901**	**0.929**	**0.878**
	DNABERT-2	0.650	0.894	0.930	0.891	0.922	0.871
	GENA-LM	0.636	0.877	0.923	0.887	0.911	0.862
	HyenaDNA	0.610	0.875	0.906	0.873	0.908	0.847
	Mistral-DNA	0.402	0.687	0.762	0.734	0.748	0.710
	Nucleotide Transformer	0.632	0.880	0.925	0.881	0.920	0.867

*Ab initio*	Probing-head-like	0.474	0.754	0.836	0.757	0.807	0.741
	ChromBPNet-like	0.667	**0.903**	0.929	0.894	0.921	0.848

We underline the best-performing model for each setting and bold the best-performing model across all settings. For each model, we evaluated overall accuracy across all classes and AUROC between each class and the remainder. We see that *ab initio* sequence models performed comparably to the best fine-tuned DNALMs and substantially outperformed all probed DNALMs.

**Table 5:** Chromatin activity prediction

		Spearman *r* among positives					AUROC (positives vs. negatives)				
Setting	Model	GM12878	H1ESC	HEPG2	IMR90	K562	GM12878	H1ESC	HEPG2	IMR90	K562
Probed	Caduceus	0.251	0.371	0.312	0.149	0.401	0.605	0.608	0.611	0.610	0.616
	DNABERT-2	0.395	0.584	0.357	0.275	0.483	0.757	0.763	0.650	0.729	0.721
	GENA-LM	0.490	0.678	0.401	0.329	0.461	0.784	0.809	0.771	0.799	0.761
	HyenaDNA	0.362	0.538	0.345	0.237	0.438	0.708	0.728	0.641	0.702	0.662
	Mistral-DNA	0.293	0.500	0.349	0.244	0.431	0.586	0.644	0.653	0.712	0.678
	NT	0.410	0.595	0.337	0.270	0.499	0.757	0.765	0.648	0.739	0.764

Fine-Tuned	Caduceus	0.503	0.744	0.454	0.479	0.570	0.935	0.954	0.896	**0.976**	0.933
	DNABERT-2	0.489	0.717	0.472	0.470	0.529	0.916	0.940	0.893	0.963	0.917
	GENA-LM	0.467	0.696	0.439	0.421	0.532	0.908	0.942	0.878	0.961	0.910
	HyenaDNA	0.435	0.672	0.406	0.426	0.446	0.853	0.927	0.854	0.941	0.750
	Mistral-DNA	0.372	0.573	0.360	0.302	0.430	0.789	0.838	0.731	0.855	0.796
	NT	0.515	0.737	0.513	0.489	**0.583**	0.938	**0.958**	**0.922**	0.975	**0.941**

*Ab initio*	ChromBPNet	**0.540**	**0.754**	**0.534**	**0.549**	0.574	**0.940**	0.952	0.910	0.975	0.917

We underline the best-performing model for each setting and bold the best-performing model across all settings. For each model, we evaluated the correlation between predicted and true signals across peak regions. Additionally, we evaluated classification performance against a positive set of high-confidence peak regions and a negative set of background sequences. We see that DNALM-derived models do not offer a consistent advantage over the *ab initio* baseline models.

**Table 6:** Variant scoring

		Zero-Shot AUROC		Probed		Fine-tuned		*Ab initio*	
Dataset	Model	Likelihood	Embedding	Pearson *r*	AUROC	Pearson *r*	AUROC	Pearson *r*	AUROC
African	Caduceus	0.525	0.519	−0.021	0.512	0.471	0.650	-	-
	DNABERT-2	-	0.480	0.017	0.502	0.390	0.638	-	-
	GENA-LM	-	0.508	−0.009	0.515	0.367	0.615	-	-
	HyenaDNA	0.486	0.515	0.030	0.566	0.389	0.584	-	-
	Mistral-DNA	-	0.520	0.012	0.502	0.145	0.510	-	-
	NT	0.525	0.519	0.020	0.525	0.459	0.623	-	-
	ChromBPNet	-	-	-	-	-	-	**0.671**	**0.772**

Yoruban	Caduceus	0.443	0.508	0.017	0.490	0.513	0.666	-	-
	DNABERT-2	-	0.505	0.024	0.476	0.500	0.649	-	-
	GENA-LM	-	0.501	0.059	0.465	0.430	0.641	-	-
	HyenaDNA	0.436	0.515	−0.042	0.467	0.358	0.499	-	-
	Mistral-DNA	-	0.475	−0.003	0.432	0.053	0.504	-	-
	NT	0.469	0.613	0.129	0.516	0.557	0.697	-	-
	ChromBPNet	-	-	-	-	-	-	**0.738**	**0.892**

We underline the best-performing model for each setting and bold the best-performing model across all settings. In zero-shot settings, allelic effects of variants were scored by measuring the difference in model-derived embeddings or likelihoods of for sequences containing each allele of the variant. We computed classification metrics between positive and control variant sets, expecting positive variants to have larger predicted allelic effects. Here, unlike the other zero-shot tasks, the likelihood setting did not substantially outperform the embedding setting. In supervised settings, for the positive variants, we computed the correlation between measured and predicted allelic effects. We also computed classification metrics (AUROC and AUPRC) relative to the positive and negativevariant sets. The *ab-initio* baseline CNN (ChromBPNet) substantially outperformed DNALM-based methods.
