# Supplementary material for: DART-Eval: A Comprehensive DNA Language Model Evaluation Benchmark on Regulatory DNA
Source: ArXiv. 2025 Aug 4:arXiv:2412.05430v2. Preprint. [Version 2] (PMC12340898)
Supplement: Supplement 1 [file NIHPP2412.05430v2-supplement-1.pdf]

Checklist

1. For all authors...
   (a) Do the main claims made in the abstract and introduction accurately reflect the paper's contributions and scope? [Yes] We believe all claims in the abstract and introduction are thoroughly addressed by the rest of the paper.
   (b) Did you describe the limitations of your work? [Yes] We describe the limitations of our benchmark, along with ideas for further improvement, in the discussion section.
   (c) Did you discuss any potential negative societal impacts of your work? [No] We do not believe this work has the potential for negative societal impacts.
   (d) Have you read the ethics review guidelines and ensured that your paper conforms to them? [Yes]
2. If you are including theoretical results...
   (a) Did you state the full set of assumptions of all theoretical results? [N/A] We have no theoretical results.
   (b) Did you include complete proofs of all theoretical results? [N/A] We have no theoretical results.
3. If you ran experiments (e.g. for benchmarks)...
   (a) Did you include the code, data, and instructions needed to reproduce the main experimental results (either in the supplemental material or as a URL)? [Yes] All information is provided in the github repo, and the sources for all datasets are provided as links in this paper.
   (b) Did you specify all the training details (e.g., data splits, hyperparameters, how they were chosen)? [Yes] All information is provided in either the Modeling section or in the Appendix.
   (c) Did you report error bars (e.g., with respect to the random seed after running experiments multiple times)? [Yes] Most of our experiments are of sufficiently high sample size, but we provide full confidence intervals for our transcription factor binding motif sensitivity task on our Synapse site, and we provide error bar values for our clustering task in the paper.
   (d) Did you include the total amount of compute and the type of resources used (e.g., type of GPUs, internal cluster, or cloud provider)? [Yes] We provide this information in section D of the appendix.
4. If you are using existing assets (e.g., code, data, models) or curating/releasing new assets...
   (a) If your work uses existing assets, did you cite the creators? [Yes] All datasets are cited appropriately at the relevant locations in the paper.
   (b) Did you mention the license of the assets? [Yes] This information is mentioned in Section B of the Appendix.
   (c) Did you include any new assets either in the supplemental material or as a URL? [Yes] Main assets include processed versions of existing datasets, and these are all linked in the paper and in our Github repo.
   (d) Did you discuss whether and how consent was obtained from people whose data you're using/curating? [N/A] Data was not directly obtained from people; thus, no consent is necessary.
   (e) Did you discuss whether the data you are using/curating contains personally identifiable information or offensive content? [N/A] No identifiable information exists in our data.
5. If you used crowdsourcing or conducted research with human subjects...
   (a) Did you include the full text of instructions given to participants and screenshots, if applicable? [N/A] We did not use crowdsourcing.
   (b) Did you describe any potential participant risks, with links to Institutional Review Board (IRB) approvals, if applicable? [N/A] We did not use crowdsourcing.
   (c) Did you include the estimated hourly wage paid to participants and the total amount spent on participant compensation? [N/A] We did not use crowdsourcing.

Appendices

A Code and Data

All our data and models can be found at `https://www.synapse.org/DART_Eval_Benchmark` unless otherwise specified. Our code can be found at `https://github.com/kundajelab/DART-Eval`.

B Extended Background

B.1 The Genome and Non-Coding Regulation

DNA, present in every cell, stores the complete set of instructions essential for life. It consists of a chain of nucleotides—adenine (A), thymine (T), guanine (G), and cytosine (C)—whose specific sequences encode functional elements. While genes, which code for proteins, are the most recognized of these elements, they constitute only a fraction of the genome. The complete DNA sequence of an organism is referred to as its genome.

Within genes, *coding* sequences specify the amino acid composition of proteins. Through the processes of transcription and translation, nucleotide triplets (codons) in these sequences are translated into amino acids, which form the building blocks of proteins.

However, in humans, coding sequences account for just around 1.5% of the genome. The remaining 98.5% includes vast regions of *non-coding* DNA, some of which play essential roles in regulating when, where, and to what extent genes are expressed. In multicellular organisms, gene activation and suppression are highly context-specific, enabling a single genome to support the development of diverse cell types across tissues and organs, each responding dynamically to internal and external signals.

Among the non-coding regions, *regulatory elements* play a crucial role in controlling gene expression according to cellular context. Unlike coding regions that directly produce proteins, these regulatory sequences contain nucleotide patterns that interact with specific DNA-binding proteins known as transcription factors (TFs). These interactions can alter the 3D structure of DNA, recruiting the molecular machinery required to activate or repress nearby genes.

Understanding non-coding regulatory elements remains challenging due to their sparse, combinatorial, and context-dependent nature. DNA-binding proteins vary in presence and behavior across different cell types, making the syntax of non-coding regulatory elements highly cell-type-specific. In this way, each gene is regulated by an array of elements, such as promoters and enhancers, each with distinct properties. Promoters are located close to the transcription start site, directly adjacent to the genes they regulate, while enhancers can reside thousands of base pairs away yet still regulate gene expression.

In summary, although non-coding regulatory elements do not produce proteins, they govern the spatiotemporal patterns of gene expression, enabling the complex regulatory landscapes that underpin cellular diversity and adaptive responses in multicellular organisms.

B.2 Deep learning models of DNA elements

In recent years, several deep learning models have been developed to learn representations of different classes of DNA elements and predict their context-specific properties and activity. These models generally fall into two categories: supervised models, which are explicitly trained to map DNA sequence to associated properties or experimental measurements of biochemical activity, and self-supervised models, which learn representations of DNA sequences without any labeled data.

Supervised deep learning models have shown impressive results in modeling various types of biological sequences. For example, they have been successfully used to predict RNA splicing, a key post-transcriptional regulatory process [21], to predict protein structure from amino acid sequences [22, 24] and to predict chromatin and transcriptional activity from regulatory sequences in

diverse cell types [7, 6, 35]. These models rely on labeled data to learn mappings from sequence to structure or functional activity.

In contrast, self-supervised learning has shown great success in training protein language models. These models capture the complex syntax of protein-coding sequences [27, 42] by training on massive protein sequence datasets without requiring explicit functional labels. Due to the high information density and conserved syntax of protein-coding DNA across species, these models have proven especially adept at learning generalized protein representations that can be fine-tuned for downstream applications such as prediction of structure, interactions and even functional properties.

Recently, self-supervised DNA language models (DNALMs) have emerged as a novel approach, extending beyond protein-coding sequences to learn representations of entire genomes [34, 13, 16, 47]. Unlike protein language models, DNALMs are trained to capture the syntax across all classes of DNA elements, including diverse types of non-coding functional elements that often encode comlex and context-dependent syntax. By modeling the full spectrum of genomic sequences, DNALMs aim to capture both coding and non-coding syntax, potentially serving as foundation models for a wide array of downstream prediction tasks, potentially reducing the need for training specialized models from scratch.

C Datasets

C.1 ENCODE candidate *cis*-regulatory elements

This dataset consists of a set of approximately 2.3 million high-confidence regulatory regions as curated by the ENCODE consortium. These regions are mainly enhancers or promoters, and they are active in at least one of a wide variety of cell types. Candidate regions were first identified by integrating cell type-specific DNAse-seq chromatin accessibility data with ChIP-seq data for the H3K27ac and H3K4me3 histone marks, which are biochemical markers associated with enhancers and promoters respectively. The final set of regions, available online on the ENCODE project website, is capped at a maximum length of 350 bp. We specifically used the cCRE list produced as part of phase IV of ENCODE, which provides an over-two-fold increase in identified cCREs from phase III. This extensive dataset serves as an ideal benchmark for evaluating language models' ability to capture essential regulatory DNA features. The dataset was downloaded from `https://www.encodeproject.org/files/ENCFF420VPZ/`. All ENCODE data is available for unrestricted use.

C.2 HOCOMOCO transcription factor binding motifs

Each transcription factor recognizes specific DNA sequence motifs. To evaluate the models' ability to identify regulatory sequence features, we analyzed each motif independently. Among available motif databases, HOCOMOCO is widely used in the research community. It compiles motifs derived from ChIP-seq and HT-SELEX data, which measure protein-DNA binding, and uses the ChIPMunk motif discovery method to generate motif sequences. Version 12 of HOCOMOCO provides position-weight matrices (PWMs) for 949 human transcription factors, encompassing 1,443 unique motifs when accounting for subtypes. Each PWM provides nucleotide probabilities at each motif position, from which we derive consensus sequences by selecting the most probable nucleotide per position. The HOCOMOCO database also groups transcription factors into families, facilitating higher-level analyses. The database was downloaded from `https://hocomoco12.autosome.org/final_bundle/hocomoco12/H12CORE/formatted_motifs/H12CORE_meme_format.meme`. HOCOMOCO data is available under the WTFPL license.

C.3 ATAC-seq and DNase-seq Peaks

The peak sets are summarized in Table S1 and Table S2. The cell-type-specific peak sets, identified by DESeq2, can be visualized in Figure S1.

ATAC-seq peaks and DNase-seq peaks are defined as regions of high chromatin accessibility in the genome. These datasets were downloaded from ENCODE. The GM12878 ATAC-seq peaks were obtained from `ENCFF748UZH`. The H1ESC ATAC-seq peaks were obtained from [35]. The HEPG2 ATAC-seq peaks were obtained from `ENCSR291GJU`. The IMR90 ATAC-seq peaks were obtained from `ENCFF243NTP`. The K562 ATAC-seq peaks were obtained from `ENCFF333TAT`. Amongst the

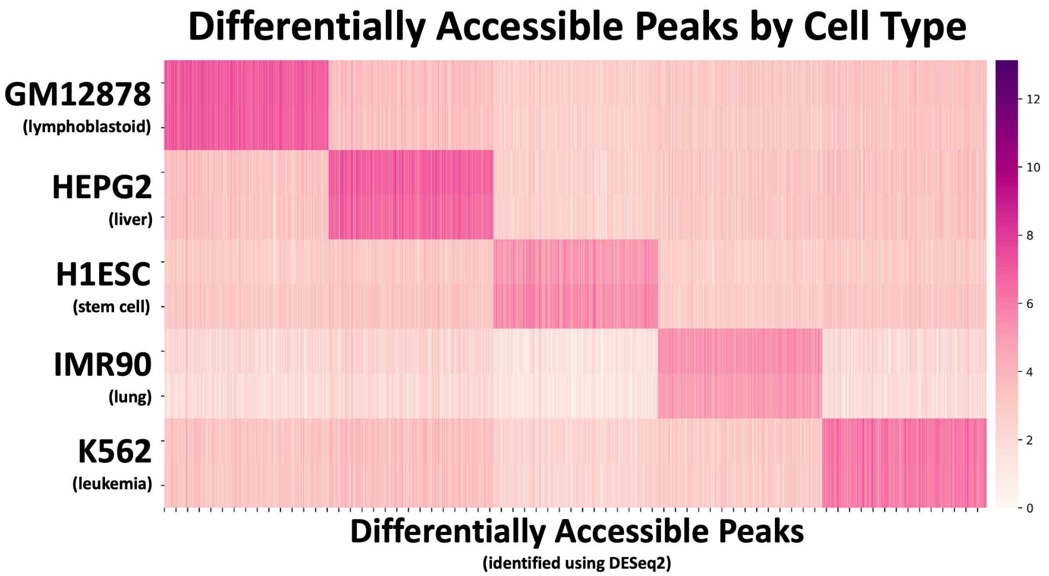

Figure S1: Correlations of per-motif embedding-based accuracies for each pair of models. Diagonals represent accuracy distribution for each model

Table S1: Overview of chromatin accessibility peak datasets used in training and evaluation: links to peaks

Cell Type	ATAC-seq Peaks	DNase-seq Raw Files
GM12878	`ENCFF748UZH`	`ENCSR000EMT`
H1ESC	[35]	`ENCSR000EMU`
HEPG2	`ENCSR291GJU`	`ENCSR149XIL`
IMR90	`ENCFF243NTP`	`ENCSR477RTP`
K562	`ENCFF333TAT`	`ENCSR000EOT`

Table S2: Overview of chromatin accessibility peak datasets used in training and evaluation: number of peaks

Cell Type	# ATAC-seq Peaks	# DNase-seq Peaks	# DNase IDR Peaks	# Differentially Accessible Peaks
GM12878	277,999	127,079	70,897	45,184
H1ESC	104,250	103,000	48,188	49,208
HEPG2	279,739	184,583	119,403	33,948
IMR90	265,247	234,313	44,807	50,783
K562	269,800	194,321	137,722	37,623

ATAC-seq datasets, there are a total of 277999 GM12878 peaks, 104250 H1ESC peaks, 279739 HEPG2 peaks, 265247 IMR90 peaks, and 269800 K562 peaks. All ENCODE data is available for unrestricted use.

The final set of DNase-seq peaks for all cell lines was obtained from [35]. The raw files were obtained from ENCODE and processed according to [35]. The GM12878 raw files were obtained from `ENCSR000EMT`. The H1ESC raw files were obtained from `ENCSR000EMU`. The HEPG2 raw files were obtained from `ENCSR149XIL`. The IMR90 raw files were obtained from `ENCSR477RTP`. The K562 raw files were obtained from `ENCSR000EOT`.

The final sets of high confidence, reproducible peaks for all cell lines were also obtained from [35].

C.4 Variants that influence chromatin accessibility (caQTLs and dsQTLs)

Molecular quantitative trait loci (QTLs) are genetic variants that influence variation of a molecular activity (e.g. gene expression or chromatin accessibility) in a particular cell type across multiple individuals. DNase-seq QTLs (dsQTLs) are genetic variants associated with variation in chromatin accessibility, as measured by DNase-seq experiments. Chromatin accessibility QTLs (caQTLs)

Table S3: Cell-Type specific motifs in differentially accessible peaks

	HOMER *-log10(q-value)*				
Motif Name	GM12878	H1ESC	HEPG2	IMR90	K562
IRF1	10	0	0	0	0
IRF2	10	0	0	0	0
SpiB	10	0	0	0	0
Oct4	0	10	0	0	0
Sox2	0	10	0	0	0
Sox6	0	10	0	0	0
Hnf4a	0	0	10	0	0
FoxA1	0	0	10	0	0
Hnf1	0	0	10	0	0
ATF3	0	0	0	10	0
Fosl2	0	0	0	10	0
Jun-AP1	0	0	0	10	0
Gata1	0	0	0	0	10
KLF4	0	0	0	0	10
Gata2	0	0	0	0	10

Table S4: Overview of variant datasets

Dataset Name	# Total Variants	# Significant Variants	# Control Variants	Original Source	Filtered Source
Chromatin QTLs in African LCLs	219,382	6,821	77,999	[15]	`syn59449898`
DNase QTLs in Yoruban LCLs	28,309	560	26,813	[14]	`syn59449898`

are genetic variants associated with variation in chromatin accessibility measured using ATAC-seq experiments. Genomic elements with strong ATAC-seq or DNase-seq signal are typically regulatory elements bound by TFs. We used two QTL datasets to evaluate all the models (Table S4). We downloaded the processed CaQTLs from [35] (File `variant_effect_benchmarking.tsv.gz` from Synapse repository `syn59449898`).

caQTLs in African LCLs. The first dataset consists of 219,382 variants and their effect sizes and statistical significance of association with variation of ATAC-seq signal across 100 lymphoblastoid cell-lines from individuals 6 African ancestry subpopulations (ESN, GWD, LWK, MSL, YRI, and MKK) [15]. After filtering the variants using the procedure described in [35], we were left with 77,999 control variants and 6,821 statistically significant caQTLs. Variants are restricted to fall within ATAC-seq peaks identified in the entire cohort in order to enrich for likely causal caQTLs. The data is available under the Creative Commons Attribution 4.0 International License.

DNase QTLs in African LCLs. We obtained a dataset from [14], which comprises 560 statistically significanct DNase I sensitivity QTL (dsQTL) variants and 26,813 control variants. We filtered the variants using the procedure described in [35]. Variants are restricted to fall within DNase-seq peaks identified in the entire cohort in order to enrich for likely causal caQTLs

D Models

D.1 Zero-Shot Model Evaluations

All pre-trained models used in this study were obtained from HuggingFace using the documentation provided in each model's README.

For all models, sequence embeddings were derived from the output of the last hidden layer when performing inference on the input sequence. Embeddings for auxiliary tokens like `<CLS>`, `<start>`, and `<end>` were removed, and the remaining embeddings were averaged to produce an overall sequence representation. For models using byte-pair encodings, where tokens represent variable numbers of nucleotides, this average is weighted by the number of nucleotides in each token. This embedding process is used in all embedding comparison tasks in this study.

To calculate model (pseudo-)likelihoods for an input sequence, obtain the predicted logits for each token. For autoregressive models, this can be done with a single forward pass, where each token is conditioned on preceding tokens. For masked models, we successively masked each token and compute predicted logits at the masked position conditioned on all other tokens. Unscaled logits

were then converted into log-likelihoods using log softmax, and the log-likelihood for the true token choice at each position is isolated. These token-level log-likelihoods were then summed across tokens (multiplied in log space) to produce the overall sequence likelihood. This sequence-level log-likelihood methodology was used for all likelihood-based comparisons in this study.

D.2 Probed and Fine-Tuned Models

For final-layer probing, the base pre-trained model weights were frozen. Outputs from the final hidden layer were passed to an additional CNN-based probing head. Embeddings were converted from token space to sequence space by repeating each token embedding by the number of nucleotides spanned by the token, as in [30] and [41]. The probing head consists of a linear projection to 32 dimensions, two convolutional layers of width 8 and 32 filters, a sum pooling layer, and a linear layer to produce the final output. ReLU activations are applied after each intermediate layer. Probing heads were trained using Adam with a learning rate of $2e^{-3}$.

Fine-tuning utilized LoRA, a widely-used parameter-efficient fine-tuning method that performs low-rank updates to model parameters [20]. For consistency across multiple architectures, we applied fine-tuning to all linear and convolutional layers. We used each model's included classifier head, trained from scratch. LoRA parameters included a rank of 8, an α of 16, and a dropout of 0.05. Optimization used AdamW with a learning rate of $1e^{-4}$ and a weight decay of 0.01.

We used a consistent train, validation, and test split across all experiments, at an approximate 4:1 train and validation to test split, and an approximate 9:1 train to validation split. Our test set consists of chromosomes 5, 10, 14, 18, 20, 22. Our validation set consists of chromosomes 6 and 21, and our training set consists of all other chromosomes. For all models, we evaluated the checkpoint with the lowest validation loss. All reported numbers were computed on the test set unless otherwise stated.

D.3 *Ab initio* Models

For the chromatin accessibility regression models - which were also used in the variant interpretation task - our *Ab initio* baseline was ChromBPNet, a convolutional neural network that can predict the magnitude and shape of chromatin accessibility profiles at base-pair resolution from an input DNA sequence. ChromBPNet takes as input a one-hot encoded DNA sequence of length 2,114, passing it through a single convolutional layer followed by 8 dilated residual layers of increasing kernel size. The output of these layers is used to make two predictions. First, a Global Average Pooling (GAP) layer is applied, followed by a linear layer to predict the total ATAC-seq or DNase-seq read counts within the central 1,000 bp of the input. Only this prediction was used to compare with the probed and fine-tuned language models. Second, the convolutional output is passed through another convolutional layer with a large kernel and only one channel, producing a predicted base-level probability profile of reads over the output region. By multiplying both model outputs together, one can obtain the predicted read counts at each position in the output region. The count prediction was trained using mean squared error loss, while the profile head was trained using log-likelihood loss based on a multinomial distribution. Separate ChromBPNet models are trained on each chromatin accessibility dataset. We utilized already trained ChromBPNet models from the ENCODE project for each dataset in this study.

For all tasks except chromatin accessibility regression and variant effect prediction, we compared against a small custom-trained CNN resembling the probing head we use, as it has a similar model capacity. This model consists of two parts: an embedding block - designed to produce simple sequence embeddings of similar dimensionality to DNALM embeddings - followed by an output head. The architecture of the output head is identical to the head used for probing. The embedding block takes in a one-hot encoded DNA sequence as input and applies a single convolutional layer of width 41 and 256 channels. This output is summed with a learned single-channel positional embedding, up-projected to 256 channels. The resulting embeddings then serve as the input to the output head. Models were trained using Adam with a learning rate of $1e^{-3}$.

For the cell type-specific regulatory DNA task, we implemented an additional larger *Ab initio* baseline resembling the ChromBPNet architecture. Differences from ChromBPNet are (1) 7 dilated residual convolutional layers instead of 8, (2) removal of the base-pair-resolution prediction head, and (3) the addition of a single-channel learned positional encoding, incorporated after the initial convolutional layer. Models were trained using Adam with a learning rate of $1e^{-4}$.

Train, validation, and test folds are identical to those used for fine-tuning and probing.

E Tasks and Results

Model training and evaluations were performed on Kundaje Lab machines, the Stanford Sherlock HPC cluster, and Google Cloud VMs. We utilized a combination of NVIDIA L40S, A100 (40 and 80 GB), V100, and Titan X GPUs depending on availability.

E.1 Distinguishing regulatory DNA from background sequences

Our first task tests whether models could discriminate regulatory elements from synthetic background sequences. For our positive set of regulatory elements, we used the ENCODE cCRE list of approximately 2.3 million high-confidence regulatory regions. We then performed dinucleotide shuffling on each cCRE sequence to produce a matched set of synthetic negative background sequences, in which negative sequences retain the same sequence composition as their positive counterparts but lack the binding motifs that promote activity. To ensure reproducibility of the shuffling process, the algorithm was seeded by the SHA-256 hash of the input region's genomic coordinates.

We then tested the models' binary classification performance in zero-shot, probed, and fine-tuned settings. In the zero-shot setting, we calculated the likelihood for each cCRE and background sequence, with a correct prediction defined as a higher likelihood for a cCRE than its corresponding background sequence. For both the probing and fine-tuned settings, we trained classifiers to predict which category a sequence belongs to.

For the zero-shot evaluation, performance metrics included accuracy and a one-sided Wilcoxon Rank-Sum Test between the cCRE and control likelihoods. For the other settings, metrics included accuracy, AUROC, and AUPRC.

E.2 Assessing sensitivity to known regulatory sequence motifs

Models were then evaluated for their ability to recognize individual transcription factor binding motifs. We used a list of 1,443 consensus transcription factor (TF) motif sequences from the HOCOMOCO v12 database. 100 neutral background sequences were randomly chosen from the cCRE classification task background set. Specifically, for each combination of neutral sequence and motif, the following sequences were considered:

1. Neutral: the original neutral sequence
2. Positive: the neutral sequence with the motif inserted at the center (for a length-n motif, the central n nucleotides of the sequence were replaced with the motif)
3. Negative: the control sequence with a shuffled version of the motif inserted at the center
4. Reverse complement of the neutral (1)
5. Reverse complement of the positive (2)
6. Reverse complement of the negative (3)

Taken together, this procedure resulted in a dataset of 577,400 unique sequences.

We employed likelihood and embedding-based approaches for this task. For the likelihood approach, we determined whether the predicted likelihood was higher for each positive sequence than for each corresponding negative sequence. 200 such pairs exist in the dataset for each motif, and we defined a model's accuracy for that motif as the proportion of pairs where the positive sequence had a higher predicted likelihood. We also utilized the results to compute a one-sided Wilcoxon Rank Sum significance test for each motif. Note that neutral sequences were not used for this analysis.

We also evaluated using an embedding-based approach with the following procedure:

1. Let $s_\square$ be a raw or reverse-complemented neutral sequence. Let s_+ be the corresponding positive sequence, and let s_- be the corresponding negative sequence.
2. We calculate d_+, the embedding distance between s_+ and $s_\square$, and d_-, the embedding distance between s_- and $s_\square$. Cosine distance is used as the embedding distance metric.

3. The prediction for the triplet $(s_+, s_-, s_\square)$ is considered correct if $d_+ > d_-$.

As with the likelihood evaluation, 200 such pairs exist per motif, allowing us to obtain an accuracy metric for each motif. Also as before, we evaluated significance using a one-sided Wilcoxon Rank Sum Test.

We additionally used metadata from the HOCOMOCO database to group motifs into motif families. We then aggregated per-motif accuracy metrics to the family level.

E.3 Learning cell-type-specific regulatory sequence features

We next evaluated whether models can discriminate accessible regulatory regions in different cell types that possess distinct sets of active sequence features. We utilized ATAC-seq peaks from five cell lines: GM12878, H1ESC, HEPG2, IMR90, and K562, with multiple biological replicates for each cell type. Details are in Appendix C.3. These cell lines are extensively studied and are also known to differ in the set of key transcription factors that regulate accessibility in each cell-line. We identified differentially-active peak sequences using DESeq2, a negative-binomial-model-derived statistical test for read-count-based experimental assays. Specifically, we formed a consensus peak set by merging and deduplicating peaks from each cell type. Then, we counted the number of ATAC reads intersecting each consensus peak region in each cell type. Then, we used DESeq2 in a one-vs-others fashion for each cell type, where the positive class corresponds to C_i, the cell type for which we are finding the differential peaks, and the negative set = $\{C_j\}$ with $j \neq i$ corresponding to all the other cell types. Our final differential peak sets were chosen with a positive log fold change > 1 and an adjusted p-value < 0.001. We only kept peaks with differential activity in exactly one cell type. We summarized the number of differentially accessible peaks in each cell type in Table S2. We validated our differential peak set using Homer [19]. HOMER is a *de novo* motif discovery algorithm that scores motifs by looking for motifs with differential enrichment between two sets of sequences. For our purposes, we used the differentially accessible peak set in one cell type as the target set and the differentially accessible peak sets in all other cell types as the background set, and we repeated this for all cell types. HOMER takes the motifs identified from the *de novo* motif discovery step and compares them against a library of known motifs in JASPAR [17]. In Table S3, we present the negative log of the Benjamini-Hochberg-adjusted q values from the HOMER motif discovery, with $-log(q)$ capped at 10.

In the zero-shot setting, we further restricted the peak sets to the top 5000 differential peaks per cell line, based on the adjusted DESeq2 p-value. On these peak sets, we produced model embeddings for each peak sequence. For the baseline, we computed motif scores using FIMO [18], which scans a collection of DNA sequences for occurrences of one or motifs from the HOCOMOCO database described in Appendix C.2. We intersected the motif hits with the peaks using BedTools [37] and constructed bag-of-motifs embeddings for each peak where each entry is the sum of the $-\log_{10}$(FIMO q-value) for a particular motif in that peak sequence. We then selected for the most variable motifs using a permutation method comparing the sum of the motif across all the peaks in each subsampled peak set. We performed the subsampling procedure 1000 times with each subsampled peak set consisting of 100 peaks. (Note that ground-truth labels are not used at any stage when constructing baseline embeddings.) We then performed k-means clusterings on each set of embeddings, with k set to 50. The ability of the clustering to differentiate peaks from different cell lines was quantified through the adjusted Mutual Information Score between the cluster labels and the true cell line labels for each peak. The Adjusted Mutual Information (AMI) score, a common method to evaluate clustering results, measures concordance between two sets of labels. Its maximum value is 1.0, with values close to 0 indicating random labeling and values close to 1 indicating a perfect match between clusters and labels. We obtained AMI scores from 100 different k-means clustering runs and define a conservative 95% confidence interval around the mean as the difference between the mean and the 2.5% quantile or the 97.5% quantile, whichever is greater.

In the probing and fine-tuning settings, we trained a five-way classifier to predict the cell line from which each peak was derived. Important metrics included accuracy, AUROC, and AUPRC.

E.4 Predicting quantitative measures of regulatory activity from sequence

This task involves predicting quantitative measurements of chromatin accessibility from sequence, quantified as DNase-Seq read counts over the sequence. DNase-Seq peaks (regions of high accessi-

bility) and count data were obtained from the ENCODE consortium for the same set of 5 cell lines used in earlier tasks: GM12878, H1ESC, HEPG2, IMR90, and K562.

ChromBPNet models were trained on the same data and used as our baseline models. We utilized the same training setup for our probing and fine-tuning models so that inputs and labels were identical to those for ChromBPNet. Specifically, the ChromBPNet preprocessing pipeline involved filtering peaks to remove read count outliers and then expanding the remaining peaks to size 2,114. In addition to accessibility peaks, ChromBPNet is also trained on matched negative genomic background sequences. Specifically, for each peak, a negative region was selected from elsewhere in the genome with the same GC content but does not fall within the peak set. The ratio of peaks to negatives in each training batch is 10:1. Within batches, half the sequences were reverse-complemented, and each sequence was shifted a maximum of 500bp in either direction, to ensure the area of highest accessibility is not always at the center of the input. The ground-truth activity for a given input sequence was defined as the number of read endpoints intersecting the central 1,000 bp.

Quantitative predictions were evaluated using the Pearson and Spearman (rank-normalized) correlation between the predicted accessibility and measured accessibility. Metrics were computed across peaks only and also across peaks and background sequences. Models were also evaluated based on their ability to classify peaks from background sequences, quantified by AUROC and AUPRC. For classification metrics, the set of positives was restricted to high-confidence, reproducible peaks, identified using the Irreproducing Discovery Rate (IDR) method [25] that determines whether peaks identified in replicate experiments are rank consistent and reproducible.

E.5 Predicting counterfactual effects of regulatory genetic variants

A critical challenge in human genetics is predicting how genetic variants affect gene regulation through changes in chromatin accessibility. Models trained to predict regulatory activity from sequence (S2A models) (such as those in Section4.4) are typically used in a counterfactual setting to predict the effects of genetic variants on regulatory activity. This is a particularly challenging task since the S2A models are never directly trained on genetic variation data. We evaluated the ability of DNALMs to prioritize and predict the quantitative effects of regulatory genetic variants that impact chromatin accessibility.

Each variant is a single nucleotide polymorphism (SNP) consisting of a pair of alleles, a reference allele $x_{ref} \in$ {A,C,G,T} and an alternate allele $x_{alt} \in$ {A,C,G,T}, together with a label $y \in$ {1,0}, indicating whether the variant is a statistically significant chromatin accessibility QTL (dsQTL or caQTL) or a background variant. All genomic variant coordinates for the caQTL dataset are based on the human reference genome version GRCh38, whereas variant coordinates for the dsQTL dataset are based on the human reference genome version GRCh37.

Each allele of a variant was scored by taking a sequence of length 2114, where the variant allele was placed in the center of a 2114-length sequence, with the remaining sequence provided as context. Both sequences, with reference and alternate alleles respectively, were passed through the model to obtain scores for each.

In the zero-shot embedding setting, given reference and alternate alleles, two embeddings were computed, and the cosine distance between the embeddings was used as the allelic effect score of the variant. In the zero-shot likelihood setting, the variant position was masked out and the likelihoods at the mask token with respect to the reference and alternate alleles are compared. In supervised settings, we evaluated the predicted counts log fold change between the two alleles.

Table S5: Resource requirements of evaluated DNALMs.

Model	Variant	Parameters	Inference Runtime (ms)	Inference Memory (GB)	Training Runtime (ms)	Training Memory (GB)
Caduceus	`ps_131k_d-256_n-16`	7,725,568	239.67 ± 1.11	1.07 ± 0.00	834.78 ± 3.67	40.82 ± 0.00
DNABERT-2	`117M`	117,069,313	104.17 ± 2.06	1.59 ± 0.03	325.93 ± 6.74	8.81 ± 0.17
GENA-LM	`bert-large-t2t`	336,658,433	194.04 ± 5.47	3.17 ± 0.02	502.53 ± 13.54	18.35 ± 0.73
HyenaDNA	`large-1m`	6,550,784	59.51 ± 0.57	0.94 ± 0.00	174.08 ± 3.77	7.57 ± 0.00
Mistral-DNA	`v1-1.6B-hg38`	1,607,677,440	129.63 ± 7.41	9.35 ± 0.03	351.36 ± 13.54	14.69 ± 0.24
Nucleotide Transformer	`v2-500m-multi-species`	494,134,738	289.58 ± 0.76	4.44 ± 0.00	733.30 ± 2.12	22.21 ± 0.00

DNALM resource requirements per batch of 64 sequences of length 2114 bp. Statistics are displayed as mean ± standard deviation. Values include each model's classification head. Gradients were computed for all model parameters when measuring training resource requirements. This evaluation was conducted on an Nvidia L40S GPU.

Table S6: Regulatory element identification extended results

Setting	Model	Absolute Accuracy	Paired Accuracy	AUROC	AUPRC
Probed	Caduceus	0.7257 ± 4.0344e-04	0.8961 ± 2.7588e-04	0.8203	0.8319
	DNABERT-2	0.8467 ± 3.2579e-04	0.9428 ± 2.1003e-04	0.9314	0.9366
	GENA-LM	<u>0.8867 ± 2.8661e-04</u>	<u>0.9594 ± 1.7857e-04</u>	<u>0.9580</u>	<u>0.9627</u>
	HyenaDNA	0.8475 ± 3.2511e-04	0.9347 ± 2.2338e-04	0.9274	0.9300
	Mistral-DNA	0.7591 ± 3.8671e-04	0.8587 ± 3.1503e-04	0.8430	0.8492
	Nucleotide Transformer	0.8194 ± 3.4785e-04	0.9168 ± 2.4980e-04	0.9025	0.9043
Fine-Tuned	Caduceus	0.9030 ± 2.6769e-04	0.9707 ± 1.5260e-04	0.9723	0.9746
	DNABERT-2	0.9131 ± 2.5467e-04	0.9730 ± 1.4650e-04	0.9745	0.9769
	GENA-LM	0.9095 ± 2.5946e-04	0.9722 ± 1.4875e-04	0.9746	0.9772
	HyenaDNA	0.8768 ± 2.9721e-04	0.9523 ± 1.9264e-04	0.9505	0.9530
	Mistral-DNA	0.8167 ± 3.4986e-04	0.9053 ± 2.6476e-04	0.9017	0.9068
	Nucleotide Transformer	<u>**0.9200 ± 2.4530e-04**</u>	<u>**0.9762 ± 1.3775e-04**</u>	<u>**0.9781**</u>	<u>**0.9804**</u>
Ab initio	Probing-head-like	0.8460 ± 3.2640e-04	0.9320 ± 2.2765e-04	0.927	0.931

Table S7: Quantiles of motif identification accuracies for each model

Setting	Model	0%	25%	50%	75%	100%
Likelihood	Caduceus	0.035	0.420	0.570	0.700	**1.000**
	DNABERT-2	0.145	**0.495**	0.590	0.685	**1.000**
	GENA-LM	0.055	0.475	0.620	0.740	**1.000**
	HyenaDNA	0.000	0.420	**0.645**	**0.820**	0.995
	Mistral-DNA	0.002	0.455	0.625	0.770	**1.000**
	Nucleotide Transformer	0.200	0.465	0.565	0.658	0.995
Embedding	Caduceus	0.370	0.475	0.500	0.525	0.630
	DNABERT-2	0.375	0.480	0.500	0.525	0.635
	GENA-LM	**0.390**	0.485	0.510	0.535	0.630
	HyenaDNA	0.370	0.480	0.505	0.530	0.610
	Mistral-DNA	**0.390**	0.475	0.495	0.520	0.635
	Nucleotide Transformer	**0.390**	0.480	0.505	0.530	0.615

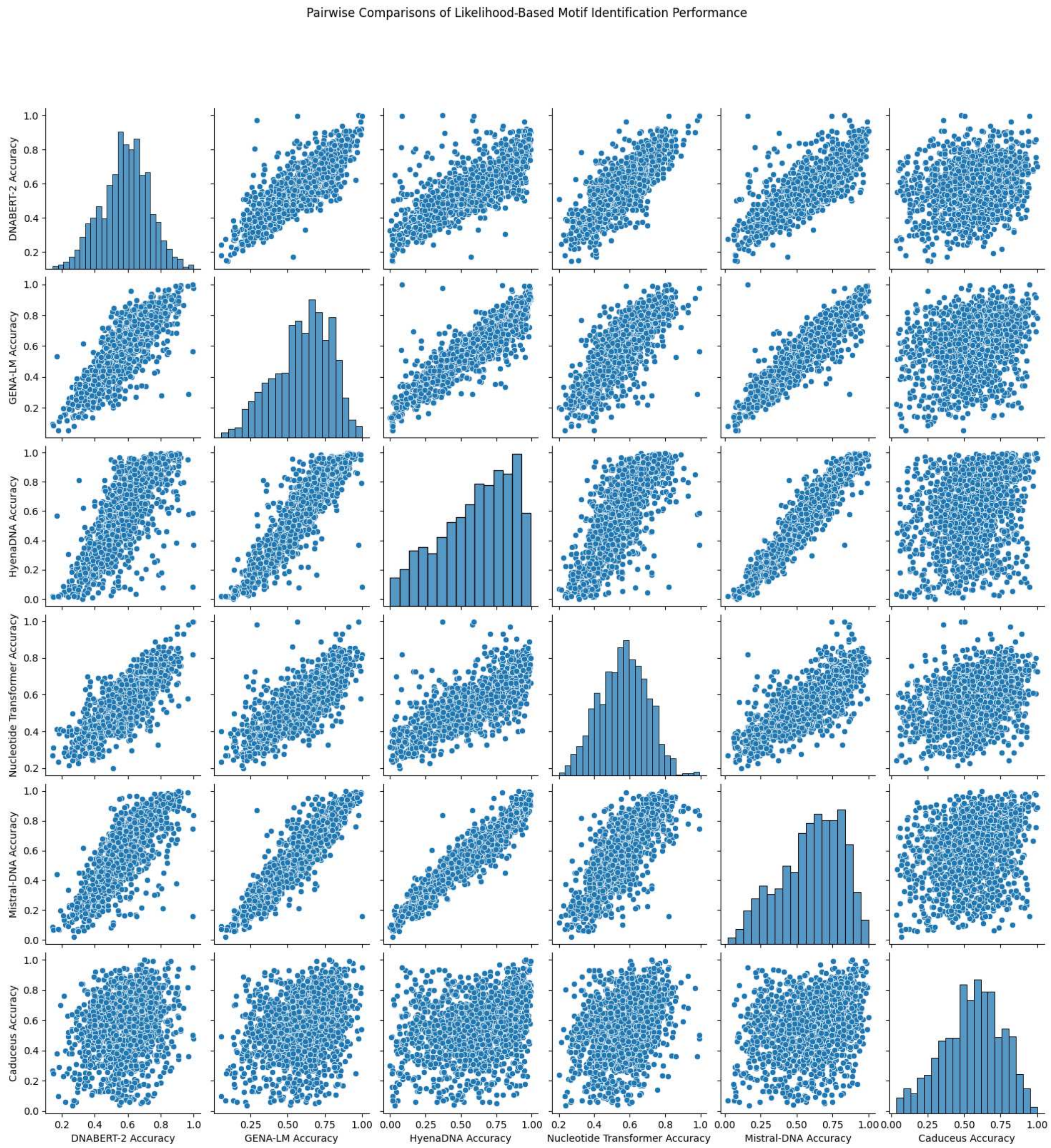

Figure S2: Correlations of per-motif likelihood-based accuracies for each pair of models. Diagonals represent accuracy distribution for each model

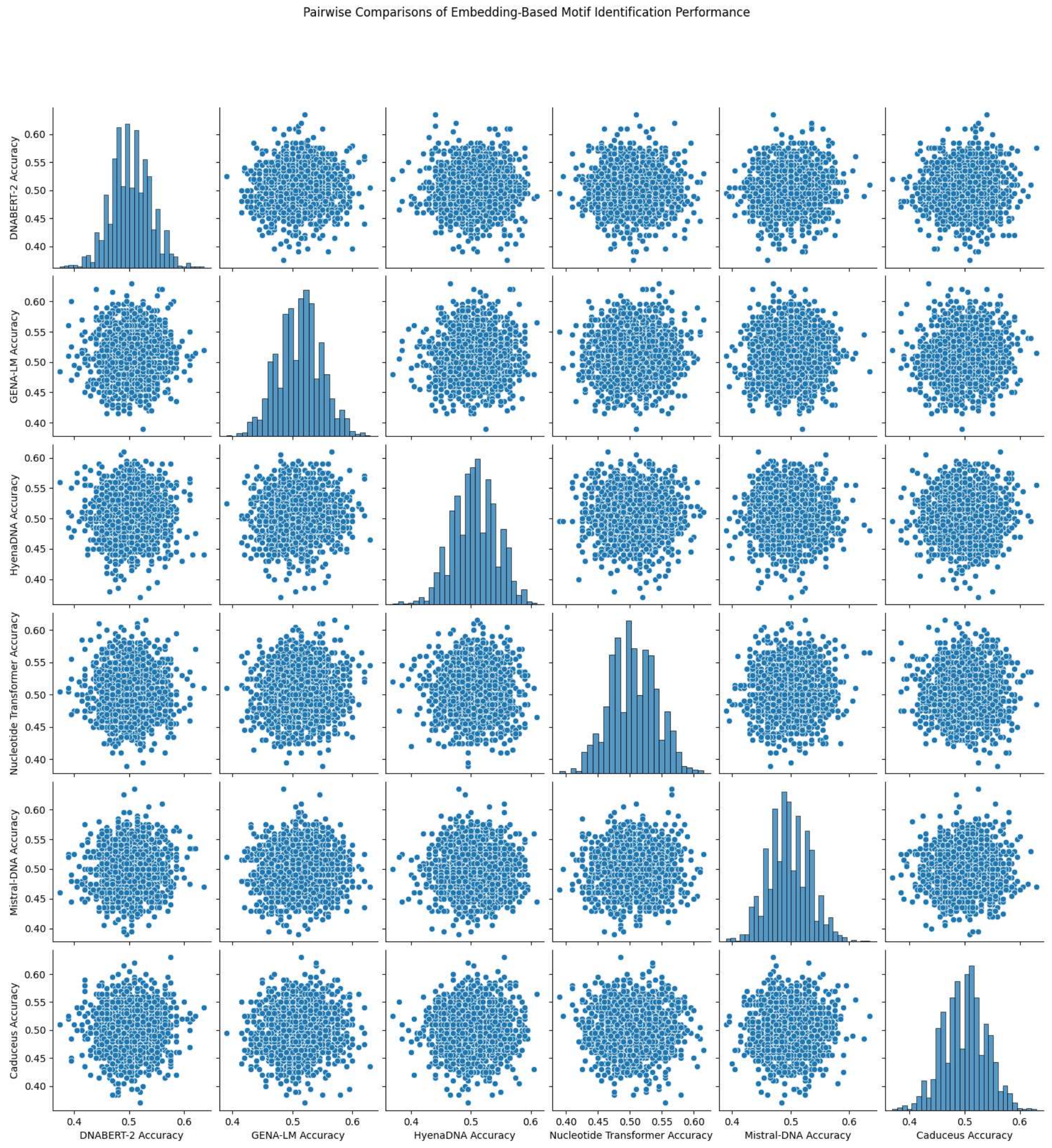

Figure S3: Correlations of per-motif embedding-based accuracies for each pair of models. Diagonals represent accuracy distribution for each model

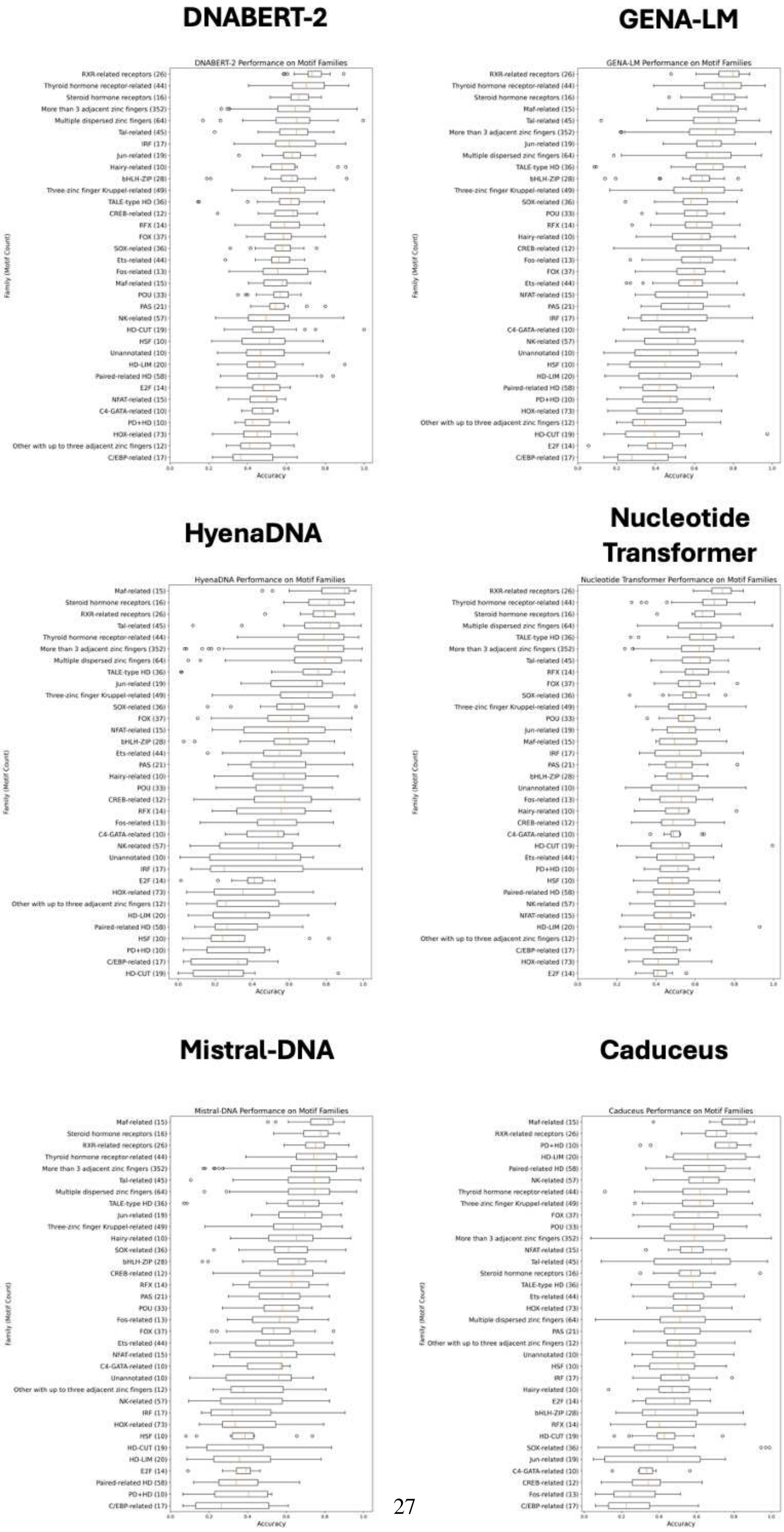

Figure S4: Likelihood-based motif detection accuracy distributions for each motif family

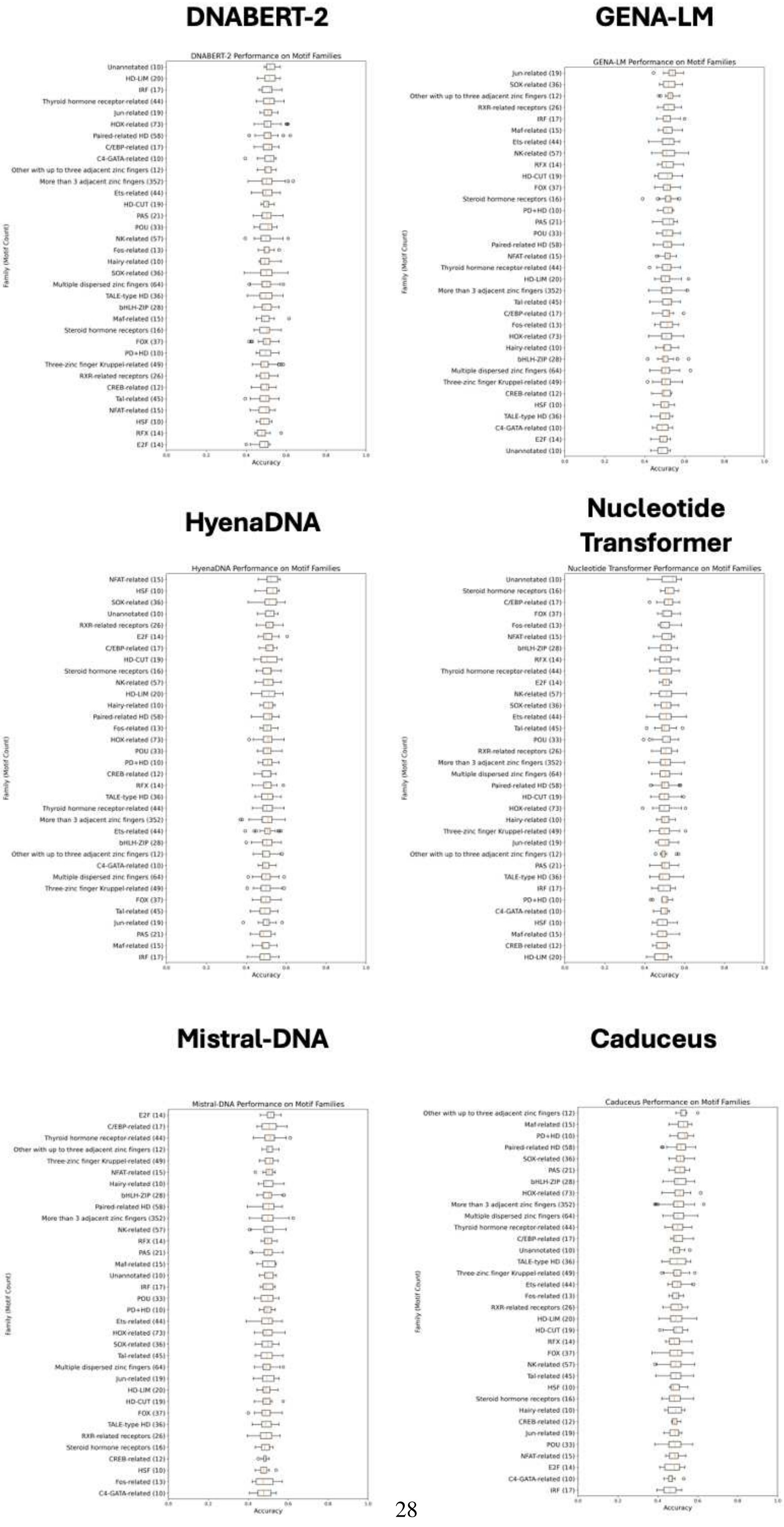

Figure S5: Embedding-based motif detection accuracy distributions for each motif family

Table S8: Cell-type specific element classification results (multi-class overall accuracy)

Setting	Model	Overall Accuracy
Probed	Caduceus	0.281 ± 1.893e-03
	DNABERT-2	0.371 ± 2.033e-03
	GENA-LM	0.383 ± 2.046e-03
	HyenaDNA	0.587 ± 2.073e-03
	Mistral-DNA	0.329 ± 1.979e-03
	Nucleotide Transformer	0.420 ± 2.078e-03
Fine-Tuned	Caduceus	**0.671 ± 1.978e-03**
	DNABERT-2	0.650 ± 2.008e-03
	GENA-LM	0.636 ± 2.025e-03
	HyenaDNA	0.610 ± 2.053e-03
	Mistral-DNA	0.402 ± 2.064e-03
	Nucleotide Transformer	0.632 ± 2.030e-03
Ab initio	ChromBPNet-like	0.667 ± 1.984e-03
	Probing-head-like	0.474 ± 2.102e-03

Table S9: Cell-type specific element classification results (GM12878 vs. rest)

Setting	Model	Accuracy	AUROC	AUPRC
Probed	Caduceus	0.7912 ± 1.7110e-03	0.5354	0.2300
	DNABERT-2	0.7891 ± 1.7173e-03	0.6516	0.3225
	GENA-LM	0.7919 ± 1.7089e-03	0.6267	0.2949
	HyenaDNA	0.8556 ± 1.4798e-03	0.8494	0.6799
	Mistral-DNA	0.7912 ± 1.7110e-03	0.5822	0.2745
	Nucleotide Transformer	0.8122 ± 1.6440e-03	0.7440	0.4857
Fine-Tuned	Caduceus	0.8854 ± 1.3411e-03	0.8998	0.7839
	DNABERT-2	0.8784 ± 1.3761e-03	0.8939	0.7654
	GENA-LM	0.8687 ± 1.4219e-03	0.8770	0.7304
	HyenaDNA	0.8662 ± 1.4332e-03	0.8755	0.7226
	Mistral-DNA	0.7966 ± 1.6947e-03	0.6871	0.3977
	Nucleotide Transformer	0.8680 ± 1.4251e-03	0.8800	0.7337
Ab initio	Probing-head-like	0.8120 ± 1.6449e-03	0.7538	0.5367
	ChromBPNet-like	**0.8865 ± 1.3355e-03**	**0.9026**	**0.7889**

Table S10: Cell-type specific element classification results (H1ESC vs. rest)

Setting	Model	Accuracy	AUROC	AUPRC
Probed	Caduceus	0.7775 ± 1.7510e-03	0.6221	0.2889
	DNABERT-2	0.7907 ± 1.7126e-03	0.7572	0.4755
	GENA-LM	0.8062 ± 1.6640e-03	0.7868	0.5473
	HyenaDNA	0.8448 ± 1.5243e-03	0.8893	0.7312
	Mistral-DNA	0.7777 ± 1.7503e-03	0.6775	0.3497
	Nucleotide Transformer	0.7962 ± 1.6958e-03	0.7953	0.5251
Fine-Tuned	Caduceus	**0.8941 ± 1.2956e-03**	**0.9370**	**0.8353**
	DNABERT-2	0.8861 ± 1.3373e-03	0.9300	0.8163
	GENA-LM	0.8806 ± 1.3653e-03	0.9229	0.7977
	HyenaDNA	0.8684 ± 1.4234e-03	0.9060	0.7612
	Mistral-DNA	0.7776 ± 1.7507e-03	0.7623	0.4759
	Nucleotide Transformer	0.8811 ± 1.3628e-03	0.9252	0.8054
Ab initio	Probing-head-like	0.8289 ± 1.5855e-03	0.8360	0.6458
	ChromBPNet-like	0.8856 ± 1.3401e-03	0.9286	0.8230

Table S11: Cell-type specific element classification results (HEPG2 vs. rest)

Setting	Model	Accuracy	AUROC	AUPRC
Probed	Caduceus	0.8167 ± 1.6290e-03	0.6801	0.3466
	DNABERT-2	0.8270 ± 1.5925e-03	0.7619	0.4490
	GENA-LM	0.8266 ± 1.5939e-03	0.7727	0.4608
	HyenaDNA	0.8586 ± 1.4668e-03	0.8620	0.6331
	Mistral-DNA	0.8165 ± 1.6297e-03	0.7235	0.4038
	Nucleotide Transformer	0.8230 ± 1.6067e-03	0.7831	0.4672
Fine-Tuned	Caduceus	**0.8823 ± 1.3567e-03**	**0.9009**	**0.7349**
	DNABERT-2	0.8750 ± 1.3923e-03	0.8910	0.7026
	GENA-LM	0.8745 ± 1.3946e-03	0.8871	0.6956
	HyenaDNA	0.8623 ± 1.4508e-03	0.8737	0.6539
	Mistral-DNA	0.8225 ± 1.6084e-03	0.7344	0.4121
	Nucleotide Transformer	0.8706 ± 1.4129e-03	0.8811	0.6797
Ab initio	Probing-head-like	0.8223 ± 1.6092e-03	0.7574	0.4163
	ChromBPNet-like	0.8739 ± 1.3975e-03	0.8938	0.7062

Table S12: Cell-type specific element classification results (IMR90 vs. rest)

Setting	Model	Accuracy	AUROC	AUPRC
Probed	Caduceus	0.7613 ± 1.7945e-03	0.5765	0.2806
	DNABERT-2	0.7618 ± 1.7934e-03	0.6907	0.3901
	GENA-LM	0.7629 ± 1.7905e-03	0.7137	0.4250
	HyenaDNA	0.8377 ± 1.5525e-03	0.8816	0.7216
	Mistral-DNA	0.7612 ± 1.7949e-03	0.6428	0.3469
	Nucleotide Transformer	0.7759 ± 1.7554e-03	0.7794	0.5197
Fine-Tuned	Caduceus	**0.8784 ± 1.3759e-03**	**0.9294**	**0.8211**
	DNABERT-2	0.8626 ± 1.4492e-03	0.9220	0.7993
	GENA-LM	0.8582 ± 1.4685e-03	0.9112	0.7811
	HyenaDNA	0.8531 ± 1.4901e-03	0.9075	0.7729
	Mistral-DNA	0.7775 ± 1.7511e-03	0.7479	0.4938
	Nucleotide Transformer	0.8586 ± 1.4669e-03	0.9204	0.7977
Ab initio	Probing-head-like	0.8049 ± 1.6683e-03	0.8065	0.5968
	ChromBPNet-like	0.8744 ± 1.3951e-03	0.9208	0.7998

Table S13: Cell-type specific element classification results (K562 vs. rest)

Setting	Model	Accuracy	AUROC	AUPRC
Probed	Caduceus	0.8533 ± 1.4897e-03	0.5873	0.1940
	DNABERT-2	0.8550 ± 1.4821e-03	0.6913	0.2951
	GENA-LM	0.8563 ± 1.4766e-03	0.6929	0.3004
	HyenaDNA	0.8573 ± 1.4726e-03	0.7991	0.4390
	Mistral-DNA	0.8560 ± 1.4782e-03	0.6456	0.2589
	Nucleotide Transformer	0.8473 ± 1.5144e-03	0.7109	0.3209
Fine-Tuned	Caduceus	0.8391 ± 1.5469e-03	**0.8776**	**0.5974**
	DNABERT-2	0.8358 ± 1.5595e-03	0.8715	0.5757
	GENA-LM	0.8361 ± 1.5585e-03	0.8622	0.5706
	HyenaDNA	0.8384 ± 1.5495e-03	0.8468	0.5333
	Mistral-DNA	0.8316 ± 1.5754e-03	0.7100	0.3239
	Nucleotide Transformer	0.8354 ± 1.5613e-03	0.8667	0.5707
Ab initio	Probing-head-like	0.8492 ± 1.5067e-03	0.7411	0.3343
	ChromBPNet-like	**0.8645 ± 1.4408e-03**	0.8475	0.4982

Table S14: Chromatin Accessibility Prediction Results (GM12878)

Setting	Model	Spearman r Peaks	Pearson r Peaks	Spearman r All	Pearson r All	AUROC	AUPRC
Probed	Caduceus	0.2510	0.3028	0.1751	0.2157	0.6053	0.4521
	DNABERT-2	0.3946	0.4625	0.4899	0.5308	0.7570	0.6399
	GENA-LM	0.4899	0.5369	0.5014	0.5572	0.7836	0.6794
	HyenaDNA	0.3619	0.4125	0.3964	0.4693	0.7082	0.5707
	Mistral-DNA	0.2932	0.2669	0.2266	0.3418	0.5858	0.3692
	Nucleotide Transformer	0.4098	0.4556	0.4780	0.5191	0.7565	0.6271
Fine-Tuned	Caduceus	0.5029	0.5596	0.7405	0.7304	0.9350	0.8724
	DNABERT-2	0.4892	0.5436	0.7304	0.7286	0.9157	0.8425
	GENA-LM	0.4669	0.5347	0.7196	0.7206	0.9084	0.8333
	HyenaDNA	0.4356	0.4962	0.6058	0.6069	0.8532	0.7452
	Mistral-DNA	0.3718	0.4368	0.5175	0.5557	0.7888	0.6694
	Nucleotide Transformer	0.5148	0.5862	**0.7659**	**0.7650**	0.9381	**0.8868**
Ab initio	ChromBPNet	**0.5401**	**0.6074**	0.7349	0.7282	**0.9399**	0.8851

Table S15: Chromatin Accessibility Prediction Results (H1ESC)

Setting	Model	Spearman r Peaks	Pearson r Peaks	Spearman r All	Pearson r All	AUROC	AUPRC
Probed	Caduceus	0.3706	0.4624	0.2484	0.3267	0.6076	0.4291
	DNABERT-2	0.5835	0.6477	0.5714	0.6035	0.7629	0.6412
	GENA-LM	0.6779	0.7074	0.6311	0.6682	0.8093	0.7036
	HyenaDNA	0.5381	0.6070	0.5154	0.5630	0.7282	0.5932
	Mistral-DNA	0.4997	0.5002	0.4370	0.4700	0.6445	0.4424
	Nucleotide Transformer	0.5945	0.6544	0.5418	0.5611	0.7654	0.6504
Fine-Tuned	Caduceus	0.7437	0.7869	0.7983	0.7992	0.9541	0.9081
	DNABERT-2	0.7173	0.7732	0.7810	0.7962	0.9405	0.8908
	GENA-LM	0.6962	0.7550	0.7768	0.7962	0.9416	0.8956
	HyenaDNA	0.6726	0.7271	0.7400	0.7486	0.9272	0.8610
	Mistral-DNA	0.5734	0.6478	0.6362	0.6835	0.8385	0.7353
	Nucleotide Transformer	0.7366	0.7969	**0.8011**	**0.8150**	**0.9584**	**0.9247**
Ab initio	ChromBPNet	**0.7549**	**0.7971**	0.7716	0.7534	0.9524	0.9062

Table S16: Chromatin Accessibility Prediction Results (HEPG2)

Setting	Model	Spearman r Peaks	Pearson r Peaks	Spearman r All	Pearson r All	AUROC	AUPRC
Probed	Caduceus	0.3123	0.3857	0.2623	0.3407	0.6108	0.5432
	DNABERT-2	0.3566	0.4241	0.3342	0.3954	0.6499	0.5736
	GENA-LM	0.4008	0.4833	0.5052	0.5558	0.7709	0.7000
	HyenaDNA	0.3453	0.3962	0.3465	0.4072	0.6414	0.5506
	Mistral-DNA	0.3487	0.4096	0.3529	0.4141	0.6528	0.5586
	Nucleotide Transformer	0.3365	0.3989	0.3175	0.3862	0.6483	0.5777
Fine-Tuned	Caduceus	0.4536	0.5234	0.6671	0.6323	0.8964	0.8219
	DNABERT-2	0.4719	0.5365	0.6858	0.6559	0.8934	0.8247
	GENA-LM	0.4392	0.5145	0.6626	0.6408	0.8777	0.8097
	HyenaDNA	0.4057	0.4782	0.6197	0.5949	0.8537	0.7732
	Mistral-DNA	0.3597	0.4241	0.4754	0.4833	0.7306	0.6331
	Nucleotide Transformer	0.5134	0.5773	**0.7184**	**0.6876**	**0.9216**	**0.8690**
Ab initio	ChromBPNet	**0.5344**	**0.6021**	0.6898	0.6711	0.9097	0.8618

Table S17: Chromatin Accessibility Prediction Results (IMR90)

Setting	Model	Spearman r Peaks	Pearson r Peaks	Spearman r All	Pearson r All	AUROC	AUPRC
Probed	Caduceus	0.1486	0.1719	0.2123	0.2163	0.6096	0.2702
	DNABERT-2	0.2745	0.2884	0.4454	0.4448	0.7285	0.4125
	GENA-LM	0.3288	0.3638	0.5213	0.5539	0.7989	0.5410
	HyenaDNA	0.2371	0.2869	0.3856	0.4408	0.7016	0.3889
	Mistral-DNA	0.2439	0.2828	0.3866	0.3956	0.7116	0.4025
	Nucleotide Transformer	0.2693	0.3184	0.4379	0.4427	0.7387	0.4795
Fine-Tuned	Caduceus	0.4793	0.5258	0.7988	0.7475	**0.9760**	0.8997
	DNABERT-2	0.4699	0.5126	0.7960	0.7505	0.9629	0.8580
	GENA-LM	0.4211	0.4778	0.7898	0.7512	0.9612	0.8569
	HyenaDNA	0.4255	0.4703	0.7231	0.6737	0.9412	0.7851
	Mistral-DNA	0.3023	0.3425	0.5912	0.5830	0.8547	0.6093
	Nucleotide Transformer	0.4890	0.5416	**0.8195**	**0.7806**	0.9745	**0.9018**
Ab initio	ChromBPNet	**0.5495**	**0.5963**	0.7749	0.7314	0.9745	0.8886

Table S18: Chromatin Accessibility Prediction Results (K562)

Setting	Model	Spearman *r* Peaks	Pearson *r* Peaks	Spearman *r* All	Pearson *r* All	AUROC	AUPRC
Probed	Caduceus	0.4006	0.5499	0.3009	0.4444	0.6164	0.5819
	DNABERT-2	0.4827	0.6215	0.4811	0.5722	0.7208	0.6701
	GENA-LM	0.4610	0.6152	0.4988	0.5811	0.7607	0.7066
	HyenaDNA	0.4381	0.5616	0.3763	0.4624	0.6621	0.5869
	Mistral-DNA	0.4307	0.5634	0.3973	0.5019	0.6782	0.6031
	Nucleotide Transformer	<u>0.4990</u>	<u>0.6339</u>	<u>0.5116</u>	<u>0.6037</u>	<u>0.7640</u>	<u>0.7203</u>
Fine-Tuned	Caduceus	0.5698	0.6668	0.7599	0.7475	0.9334	0.8852
	DNABERT-2	0.5286	0.6484	0.7357	0.7329	0.9172	0.8674
	GENA-LM	0.5323	0.6392	0.7349	0.7389	0.9096	0.8603
	HyenaDNA	0.4456	0.5878	0.5112	0.5693	0.7499	0.6729
	Mistral-DNA	0.4305	0.5678	0.5615	0.6005	0.7956	0.7117
	Nucleotide Transformer	<u>**0.5829**</u>	<u>**0.6863**</u>	<u>**0.7764**</u>	<u>**0.7714**</u>	<u>**0.9412**</u>	<u>**0.9016**</u>
Ab initio	ChromBPNet	0.5741	0.6687	0.7200	0.7246	0.9167	0.8762

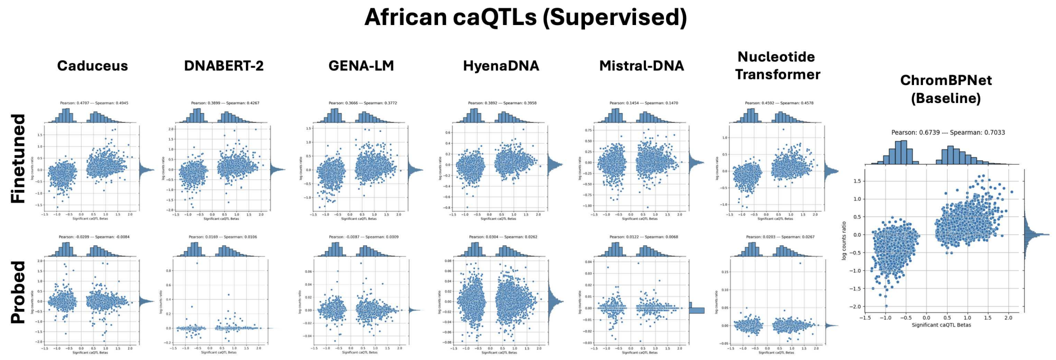

Figure S6: African LCLs caQTLs Supervised Model Scores

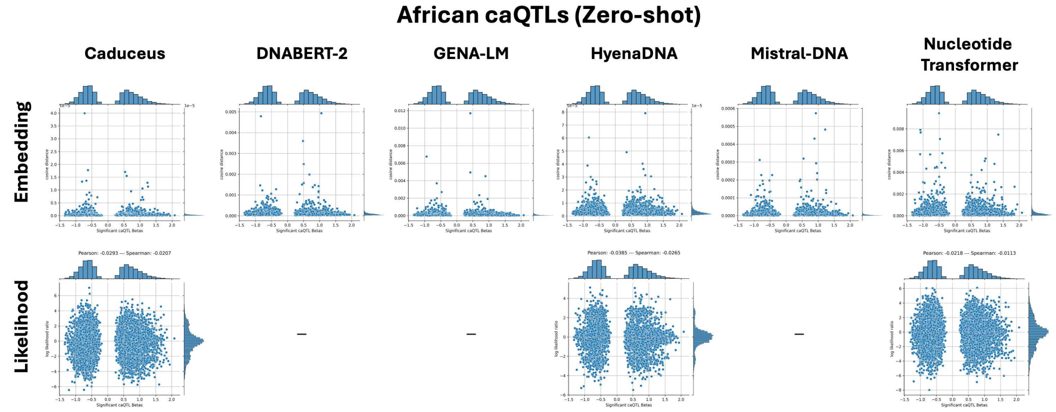

Figure S7: African LCLs caQTLs Zero Shot Model Scores

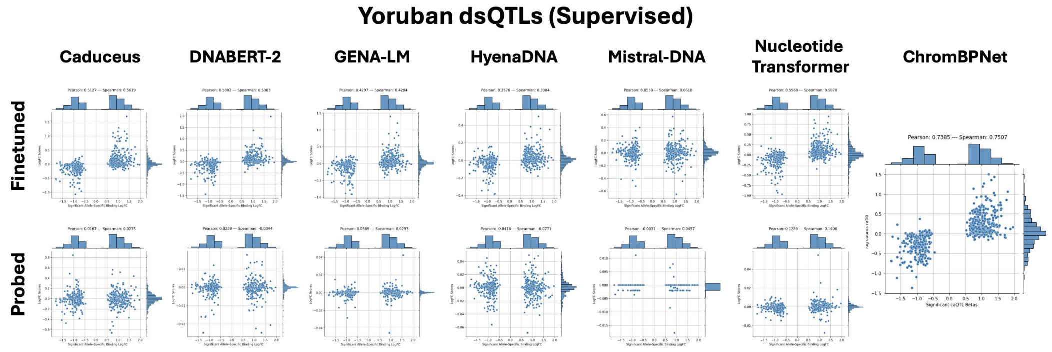

Figure S8: Yoruban LCLs dsQTLs Supervised Model Scores

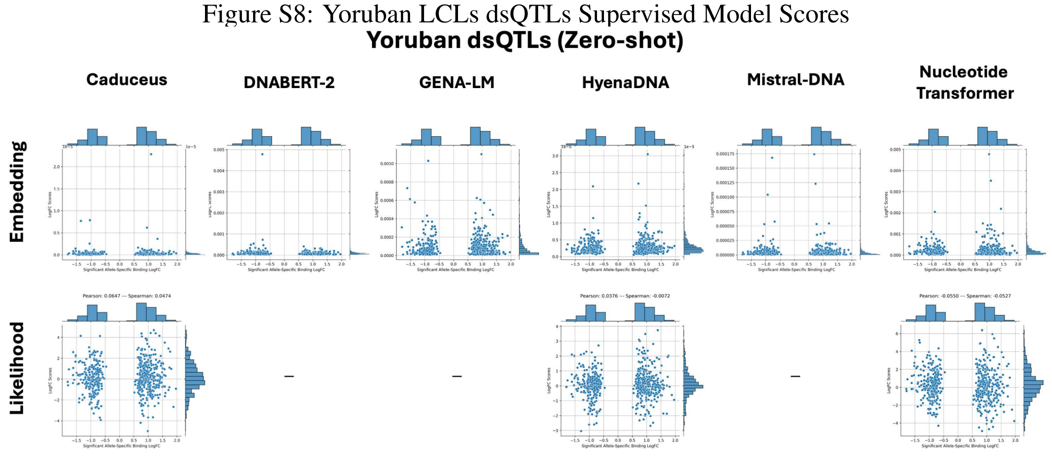

Figure S9: Yoruban LCLs dsQTLs Zero Shot Model Scores

Table S19: African caQTL Supervised Variant Scoring Extended Results

Setting	Model	Pearson *r*	Spearman *r*	AUROC	AUPRC	Wilcoxon *p*-value
Probed	Caduceus	-0.0209	-0.0084	0.5124	0.0848	0.0003
	DNABERT-2	0.0169	0.0106	0.5024	0.0810	0.2562
	GENA-LM	-0.0087	0.0009	0.5149	0.0836	2.263e-05
	HyenaDNA	0.0304	0.0262	0.5658	0.0937	3.920e-73
	Mistral-DNA	0.0122	0.0068	0.5018	0.0821	0.2706
	Nucleotide Transformer	0.0203	0.0267	0.5248	0.0870	5.336e-12
Fine-Tuned	Caduceus	0.4707	0.4945	0.6498	0.1791	**0.000**
	DNABERT-2	0.3899	0.4267	0.6385	0.1524	**0.000**
	GENA-LM	0.3666	0.3772	0.6147	0.1384	1.5595e-217
	HyenaDNA	0.3892	0.3958	0.5845	0.1060	3.9080e-119
	Mistral-DNA	0.1454	0.1470	0.5101	0.0841	0.0027
	Nucleotide Transformer	0.4592	0.4578	0.6225	0.1527	4.5174e-248
Ab initio	ChromBPNet	**0.6739**	**0.7033**	**0.7716**	**0.3972**	**0.000**

Table S20: Yoruban dsQTL Supervised Variant Scoring Extended Results

Setting	Model	Pearson *r*	Spearman *r*	AUROC	AUPRC	Wilcoxon *p*-value
Probed	Caduceus	0.0167	0.0236	0.4901	0.0200	0.7893
	DNABERT-2	0.0239	-0.0044	0.4758	0.0194	0.9756
	GENA-LM	0.0589	0.0293	0.4655	0.0191	0.9975
	HyenaDNA	-0.0416	-0.0771	0.4672	0.0187	0.9961
	Mistral-DNA	-0.0031	0.0457	0.4324	0.0201	1.000
	Nucleotide Transformer	0.1289	0.1406	0.5163	0.0222	0.0924
Fine-Tuned	Caduceus	0.5127	0.5619	0.6664	0.0764	8.176e-42
	DNABERT-2	0.5002	0.5303	0.6491	0.0556	5.239e-34
	GENA-LM	0.4297	0.4294	0.6409	0.0543	1.450e-30
	HyenaDNA	0.3576	0.3304	0.4992	0.0221	0.5272
	Mistral-DNA	0.0530	0.0618	0.5041	0.0204	0.3697
	Nucleotide Transformer	0.5569	0.5870	0.6970	0.0816	8.547e-58
Ab initio	ChromBPNet	**0.7385**	**0.7507**	**0.8916**	**0.3587**	**7.610e-222**

Corrigendum (July 31, 2025):

We have identified and corrected inconsistencies in the variant effect prediction evaluation (Task 5) that resulted from two distinct issues:

1. **Allele orientation**: A subset of African caQTLs had swapped reference and alternate alleles, causing incorrect effect size signs for sign-dependent evaluation metrics.
2. **Peak region definitions**: Chromatin accessibility peak definitions changed during DART-Eval development and were corrected for Task 4 prior to publication but not for Task 5.

After recomputing all metrics, the key changes are:

- **African caQTL dataset**: An improvement in Pearson and Spearman correlations for fine-tuned DNALMs
- **Both datasets**: Minor changes in remaining metrics across most models

We have updated manuscript Tables 6, S19, and S20, as well as Figures S6 and S8. Complete before/after comparisons are provided here in Tables 1 and 2. Our code and data repositories have been updated accordingly.

The manuscript text remains unchanged, and the paper's conclusions remain consistent with the corrected results.

We thank Shawn Sidbon and Soumya Kundu for helping identify these issues.

Table 1: African caQTL Supervised Variant Scoring: Changed Metrics

		Pearson r		Spearman r		AUROC		AUPRC		Wilcoxon p-value	
Setting	Model	Before	After	Before	After	Before	After	Before	After	Before	After
Probed	Caduceus	-0.0044	-0.0209	0.0040	-0.0084	0.5124	0.5124	0.0848	0.0848	0.0003	0.0003
	DNABERT-2	0.0064	0.0169	0.0111	0.0107	0.5024	0.5024	0.0810	0.0810	0.2536	0.2562
	GENA-LM	-0.0067	-0.0087	0.0082	0.0009	0.5149	0.5149	0.0836	0.0836	2.2893e-5	2.263e-5
	HyenaDNA	0.0121	0.0304	0.0139	0.0262	0.5658	0.5658	0.0937	0.0937	4.0326e-73	3.920e-73
	Mistral-DNA	0.0185	0.0122	0.0142	0.0068	0.5018	0.5018	0.0821	0.0821	0.2706	0.2706
	Nucleotide Transformer	0.0058	0.0203	0.0052	0.0267	0.5248	0.5248	0.0870	0.0870	5.4018e-12	5.336e-12
Fine-Tuned	Caduceus	0.2591	0.4707	0.2818	0.4945	0.6498	0.6498	0.1791	0.1791	0.000	0.000
	DNABERT-2	0.1840	0.3899	0.2185	0.4267	0.6155	0.6385	0.1380	0.1524	1.1660e-220	0.000
	GENA-LM	0.2011	0.3666	0.2161	0.3772	0.6038	0.6147	0.1285	0.1384	1.2926e-178	1.5595e-217
	HyenaDNA	0.2653	0.3892	0.2871	0.3958	0.6108	0.5845	0.1233	0.1060	4.3580e-203	3.9049e-119
	Mistral-DNA	0.0849	0.1454	0.0858	0.1470	0.5018	0.5018	0.0821	0.0821	0.2706	0.2706
	Nucleotide Transformer	0.2299	0.4592	0.2435	0.4578	0.6231	0.6225	0.1542	0.1527	2.8400e-250	4.5234e-248
Ab initio	ChromBPNet	0.6712	0.6739	0.6995	0.7033	0.7716	0.7716	0.3972	0.3972	0.000	0.000

Table 2: Yoruban dsQTL Supervised Variant Scoring: Changed Metrics

		Pearson r		Spearman r		AUROC		AUPRC		Wilcoxon p-value	
Setting	Model	Before	After	Before	After	Before	After	Before	After	Before	After
Probed	DNABERT-2	0.0240	0.0239	-0.0049	-0.0044	0.4756	0.4758	0.0194	0.0194	0.9760	0.9756
	GENA-LM	0.0589	0.0589	0.0292	0.0293	0.4655	0.4655	0.0191	0.0191	0.9975	0.9975
	HyenaDNA	-0.0417	-0.0416	-0.0771	-0.0771	0.4672	0.4672	0.0187	0.0187	0.9961	0.9961
	Nucleotide Transformer	0.1289	0.1289	0.1407	0.1406	0.5163	0.5163	0.0222	0.0222	0.0934	0.0924
Fine-Tuned	DNABERT-2	0.4727	0.5002	0.5007	0.5303	0.6307	0.6491	0.0416	0.0556	1.390e-26	5.239e-34
	GENA-LM	0.4141	0.4297	0.4541	0.4294	0.6280	0.6409	0.0396	0.0543	1.524e-25	1.450e-30
	HyenaDNA	0.5031	0.3576	0.5011	0.3304	0.5729	0.4992	0.0289	0.0221	1.6439e-09	0.5271
	Nucleotide Transformer	0.5073	0.5569	0.5223	0.5870	0.6697	0.6970	0.0796	0.0816	1.949e-43	8.547e-58

Note: Results for Caduceus, Mistral-DNA, and ChromBPNet are unchanged for the Yoruban dsQTL data.
